# Structure and Compressional Behavior of Sillén-Type (BiO)_2_(CO_3_) Bismutite Carbonate

**DOI:** 10.1021/acs.inorgchem.6c01891

**Published:** 2026-07-24

**Authors:** Raquel Chuliá-Jordán, David Santamaría-Pérez, Benedito Donizeti Botan-Neto, Ganesh Bera, Julio Pellicer-Porres, Alberto Otero-de-la-Roza, Lkhamsuren Bayarjargal, Julia Bungarten, Catalin Popescu, Bihan Wang

**Affiliations:** † Departamento de Didáctica de las Ciencias Experimentales y Sociales, Universitat de Valencia, 46022 Valencia, Spain; ‡ Departamento de Física Aplicada-ICMUV, Universitat de València, MALTA Consolider Team, 46100 Valencia, Spain; § Departamento de Química Física y Analítica, Facultad de Química, Universidad de Oviedo, MALTA Consolider Team, 33006 Oviedo, Spain; ∥ Institute of Geosciences, 9173 Goethe University Frankfurt, Frankfurt 60438, Germany; ⊥ CELLS-ALBA Synchrotron Light Facility, Cerdanyola del Vallés, 08290 Barcelona, Spain; # Deutsche Elektronen-Synchrotron DESY, Notkestraße 85, Hamburg 22607, Germany

## Abstract

In this joint experimental/computational work, we study the ambient-conditions structure of bismutite (BiO)_2_(CO_3_) and its evolution under high pressure by means of a combination of in situ synchrotron X-ray diffraction (XRD) measurements, Raman spectroscopy, and density-functional theory (DFT) calculations. Using single-crystal XRD, (BiO)_2_(CO_3_) is determined to be an orthorhombic *Cmcm* phase with disorder arising from carbonate rotations at ambient-conditions and DFT confirms the stability of the structure. In situ high-pressure synchrotron powder XRD measurements showed that the *Cmcm* ambient-pressure structure adequately accounts for the observed diffraction patterns up to 17.7 GPa. The compressibility was determined, obtaining an experimental bulk modulus of 67.2(11) GPa and significant anisotropy in the axial compressibilities: the *a* and *c* axes, parallel to the (BiO)_2_^2+^ layers, are less compressible than the *b* direction perpendicular to those layers, which we justify on the basis of the bismuth lone electron pair compressibility and the slight reorientation of the carbonate groups. Raman spectroscopy measurements at ambient conditions and under pressure were used to characterize the vibrational properties of this carbonate, unvealing a differentiated behavior of the nonequivalent carbonate units.

## Graphical abstract

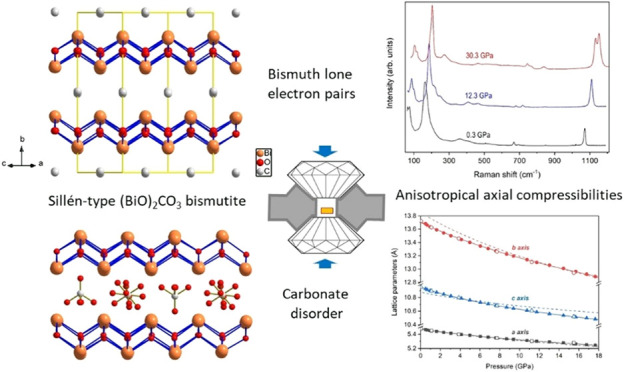

## Introduction

Bismutite is a bismuth subcarbonate mineral with formula (BiO)_2_(CO_3_) that occurs as an oxidation product of other bismuth minerals such as Bi_2_S_3_ bismuthinite and native bismuth in hydrothermal veins and pegmatites.^1^ Its chemical formula indicates that this bismuth subcarbonate contains large (BiO)_2_^2+^ dibismuthyl cations, with bismuth in oxidation state 3+ and a lone electron pair. The role of lone pair distortions in structural chemistry has been studied in numerous crystalline compounds.^2–5^ It is known that bonding pairs and lone pairs are of similar importance in defining the final topology of a compound, the lone pairs distributing themselves to minimize electrostatic repulsive interactions.^6–8^ Compounds with the (BiO)_2_^2+^ oxycations are known as Aurivillius and Sillen phases^9,10^ and are layered structures containing Bi–O sheets. Such frameworks are especially suitable for the sequestration of multiple contaminants due to their low human and environmental toxicity and cost-effectiveness.^11^ The layered crystalline arrangements of these compounds also possess promising properties because of their electronic and spatial flexibility and their ability to accommodate a wide range of negatively charged species.^11^

The determination of the structure of bismutite has not been exempt of difficulties and controversy. Originally, Lagercrantz and Sillen described (BiO)_2_(CO_3_) from X-ray diffraction (XRD) data to be tetragonal (Space Group, S.G. *I*4/*mmm*, *Z* = 2) with cell dimensions: *a* = 3.867(4) Å and *c* = 13.686(14) Å.^12^ These authors were able to determine the general location of the large Bi cations and the oxygen atoms not belonging to the carbonate groups, which together form (BiO)^+^ sheets of the same type and dimensions as those found in tetragonal bismuth oxyhalides.^13^ Unfortunately, they could not define the placement of the [CO_3_] groups themselves. In order to avoid too short O–O distances between neighboring carbonate groups placed between (BiO)^+^ sheets, the authors suggested that these groups could rotate into two perpendicular orientations and tentatively located the 6 O atoms of the [CO_3_] groups within the unitcell in a 16m Wyckoff position, with fractional occupation.^12^ Later, powder neutron diffraction investigations indicated a more complex structure and an orthorhombic unit cell with larger lattice parameters was considered (S.G. *Pna*2_1_, *a*′ = 5.468(1) Å ∼ $\sqrt{2a}$ , *b*′ = 27.32(2) Å ∼ 2*b*, *c*′ = 5.468(1) Å ∼ $\sqrt{2a}$ , *Z* = 8).^14^ However, the orientation of the [CO_3_] groups could not be properly established. Their structural model did not yield realistic thermal parameters and O–C–O bond angles, and the authors suggested that the true carbonate group representation likely required an even larger unit cell. Subsequently, Grice from single-crystal XRD data pointed to a flaw in previous deductions regarding the orientation of the [CO_3_] groups and stated that the interlayer spacing enables the accommodation of these groups.^15^ This author described the crystal structure of bismutite with the orthorhombic space group *Imm*2 and similar lattice parameters as those reported by Lagercrantz and Sillen; that is, *a*″ = 3.865(2) Å ∼ *a*, *b*″ = 3.862(2) Å ∼ *a*, *c*″ = 13.675(6) Å ∼ *c*. Huang et al. in a later work reported Rietveld refinements in agreement with the *Imm2* model.^16^ Note, however, that an analysis of the topology and distances of the latter structure shows highly distorted [CO_3_] groups (two C–O bond lengths of 1.20 Å and one C–O bond length of 1.42 Å) and extremely short O–O interatomic distances between carbonate units (1.6 Å), which points to inconsistencies in the structural solution.

At high temperatures, the bismuth subcarbonate in air, He, N_2_ and CO_2_ atmospheres (1 atm) decomposes directly into monoclinic Bi_2_O_3_ and CO_2_, with only one mass loss observed on differential thermal analysis/thermogravimetry (DTA – TG) curves at 380 °C (at a heating rate of 15 °C/min).^17^ The results in high-pressure CO_2_ atmosphere show that the decomposition temperature increases with CO_2_ pressure and that two endothermic peaks appear above P_CO2_ = 17 atm. No intermediate phase that could explain these two peaks was experimentally found in quenched samples immediately after the first decomposition peak. Besides the dependence of (BiO)_2_(CO_3_) thermal behavior on CO_2_ gas pressure, it has also been reported a decrease of the decomposition temperature with slower heating rate,^18^ as observed in other carbonates.^19^

Regarding the high-pressure (HP) structural behavior of (BiO)_2_(CO_3_), as far as we know, there are no published studies up to date. As shown below, the evolution of the structure under compression is related to the reduction of the interlayer spacing and the possible distortion of the charge density of the bismuth lone electron pair (LEP), as occurs in other group XV sesquioxides.^3,20,21^ This joint experimental and theoretical work explores the structural stability and evolution, as well as on the role played by the cationic LEP. In situ HP synchrotron X-ray diffraction (XRD) measurements provide insights into intrinsic mechanical properties of (BiO)_2_(CO_3_) such as compressibility or anisotropy, while Raman spectroscopy measurements give details of the vibrational properties. DFT calculations, on the other hand, complement experimental results and give us information about the energy of the potential polymorphs of this compound, suggesting the most stable structures over the whole range of pressures considered.

## Experimental Details

Commercial polycrystalline white bismuth subcarbonate (BiO)_2_(CO_3_) (ThermoFisher GmbH, >98.5%) was characterized at ambient conditions by means of powder XRD measurements using a Bruker D8 Advance diffractometer (Cu Kα radiation). XRD results confirmed that the sample had a pattern similar to those of previously reported structures of this compound. The ambient conditions structure of bismutite is discussed later.

Bismutite is rarely found as a single crystal. The aforementioned fine powder was subjected to high-pressure and high-temperature conditions in an attempt to achieve recrystallization. The application of pressure was intended to inhibit the thermal decomposition of the carbonate during heating, thereby stabilizing the material throughout the treatment process. Thus, bismutite powder was loaded in a Boehler-Almax-type diamond-anvil cell (DAC) of 90° opening angle to completely fill a 160-μm-diameter hole in a rhenium gasket, which had been preindented to a thickness of 50 μm. Pressure was subsequently increased to approximately 10 GPa, followed by heating at 2000 K with a CO_2_ laser until crystallization of bismutite was achieved. The temperatures during the laser heating were determined by pyrometry. We estimate an uncertainty of ∼10% of the nominal temperature in the laser-heated region due to temperature gradients. The resulting material consisted of a polycrystalline aggregate of bismutite containing crystalline domains smaller than ∼1 × 1 μm^2^. Subsequently, bismutite was quenched to ambient conditions (i.e., room temperature and ambient pressure), and XRD was then measured at P02.2 beamline at DESY using monochromatic synchrotron radiation (λ = 0.2904 Å).^22^ The beam size on the sample was approximately 2 × 2 μm^2^ (FWHM), focused by Kirkpatrick Baez mirrors. Diffraction patterns were acquired using a PerkinElmer XRD1621 detector, with a detector-to-sample distance of 400 mm. During data acquisition, the sample was rotated by ±40° around the axes perpendicular to the beam, with frames recorded in 0.5° steps and an exposure time of 0.5 s per frame. The detector-to-sample distances were calibrated with a CeO_2_ standard using DIOPTAS.^23^ The diffractometer and detector geometry were calibrated by measuring a single-crystal of enstatite (MgSiO_3_). After the measurement, the reflections were indexed and integrated using the CrysAlis^Pro^ software version 171.44.122a.^24^ The Domain Auto Finder (DAFi) program^25^ was employed to identify and sort crystalline domains within the multigrain sample, enabling the integration of reflections from the desired phase. The subsequent structural solution and refinement were carried out by Olex2,^26^ which used SHELXT^27^ and SHELXL^28^ for crystal structure determination and refinement, respectively. X-ray diffraction mapping was performed over a 110 × 110 μm^2^ (width × height) grid, with a step size of 4 μm and an acquisition time of 1 s per spectrum in order to identify the regions exhibiting the highest diffraction quality for single-crystal measurements. Data from three selected regions were merged to maximize the number of unique reflections. The structure was solved using data with a minimum resolution of 0.6 Å.

As the crystallite size could not be measured directly, the crystalline domain size was estimated from the distribution of the full width at half-maximum (FWHM) values of several bismutite diffraction peaks extracted from the XRD mapping data. Assuming that the observed peak broadening originated predominantly from the sample after correction for instrumental broadening, the crystallite size was estimated using the Scherrer equation.^29^ The crystallite-size distribution yielded an average crystallite size of approximately 0.25(2) μm, suggesting that the polycrystalline aggregate consists of on the order of thousand individual grains. As an independent estimate of the size of the measured crystal, a second approach was adopted based on the assumption that the diffracted intensity is proportional to the crystal volume. The indexed crystal structure accounted for approximately 2% of the total measured diffraction intensity. Under this assumption, the characteristic size is estimated to be on the order of 1 μm^3^.

We performed HP XRD experiments of the commercial polycrystalline sample at room temperature conditions using a gas membrane-driven DAC equipped with 400 μm culet-diameter diamonds. The (BiO)_2_(CO_3_) sample was placed together with a ruby chip at the center of a 200 μm diameter hole in the rhenium gasket, preindented to a thickness of 45 μm. Subsequently, high-purity neon (Ne) gas was loaded in the DAC at RT using the Sanchez Technologies gas loading apparatus. Ne acted as a quasi-hydrostatic medium in the pressure range of the study.^30,31^ The shift of the R1 ruby fluorescence peak was used as a pressure gauge, which provided pressure accuracies of 0.1 GPa in the studied range.^32^ The equation of state (EOS) of Ne was used as a second pressure gauge^30^ above 5 GPa, with the pressure difference with respect to that of the ruby standard being <1%. In situ angle-dispersive XRD measurements under compression were taken at the synchrotron-based MSPD beamline at the ALBA-CELLS synchrotron using an X-ray wavelength of 0.4246 Å.^33^ The beamline is equipped with Kirkpatrick-Baez mirrors, focusing the X-ray beam to ∼20 μm × 20 μm, and with a Rayonix CCD detector. A precise calibration of the parameters of the detector was developed with reference LaB_6_ powder, and integration to conventional 2θ intensity data was performed with the DIOPTAS software.^23^ The indexing and refinement of the powder patterns were performed using the Unitcell,^34^ Powdercell^35^ and Fullprof^36^ program packages.

For high-pressure Raman spectroscopic measurements, we used a Boehler-Almax DAC equipped with 350 μm culet-diameter diamonds. In this case we used helium (He) as hydrostatic pressure medium.^30,31^ Measurements were carried out using a custom-built setup,^37,38^ consisting of a grating spectrometer (Princeton Instruments Acton SpectraPro SP-2356) coupled to a Pixis 256E CCD detector and a Mitutoyo microscope objective, using a Oxxius LCX-532S Nd:YAG laser (λ = 532.14 nm) as the excitation source. The spectra were calibrated using a Hg­(Ar) lamp. The system is equipped with a set of optical filters to adjust the laser power according to the sample and experimental conditions. For the experiment, we used a Raman laser spot size on the sample of ∼6 μm while using a laser power of 26 mW on the sample, in order not to damage it. The Raman spectra were measured in the range 68–1200 cm^–1^. The experimental setup also includes a laser heating system (used for the bismutite crystallization), consisting of a Coherent Diamond K-250 pulsed CO_2_ laser (λ = 10.6 μm), which was focused onto the sample from both sides. The laser beam was incident at an angle of approximately 20° with respect to the normal direction of the DAC.

## Computational Details

Density-functional theory calculations were carried out using the projector augmented wave (PAW) method^39^ implemented in Quantum ESPRESSO, version 6.5.^40^ The PBEsol functional^41^ was used with a 100 Ry cutoff for the Kohn–Sham state and a 1000 Ry cutoff for the electron density plane-wave expansion. PAW data sets from the pslibrary^42^ were employed for all atoms, with 15 (Bi), 4 (C), and 6 (O) valence electrons. We used k-point grids fine enough to ensure a convergence in the total energy of at least 0.1 mRy: 9 × 9 × 5 (*Imm*2), 5 × 1 × 5 (*Pna*2_1_), 5 × 5 × 2 (*P*4_2_ *nm*), 5 × 2 × 2 (*Pc*). *P*4_2_ *nm* is an ordered version of the *I*4/*mmm* structure, and the *Pc* structure is an ordered variant of the *Cmcm* phase determined in our single-crystal XRD experiments (see below). Geometry relaxations were carried out with strict convergence thresholds (10^–5^ Ry in the energy, 10^–4^ Ry/bohr in the forces) at zero and 50 GPa. The resulting equilibrium volumes at those pressures were used to set up a uniform volume grid, where constant-volume geometry relaxations were carried out for each phase. The resulting energy-volume curves were fitted using average strain polynomial equations of state using the gibbs2 program.^43,44^

The dynamical stability of some structures was examined by calculating phonon frequencies on a reciprocal space grid using the density-functional perturbation theory (DFPT) method implemented in Quantum ESPRESSO.^45^ Raman spectra were calculated using the coupled-perturbed Kohn–Sham method^46,47^ implemented in CRYSTAL17.^48^ For the Raman calculations, constant-volume geometry relaxations were carried out using the B3LYP functional^49,50^ in combination with the pob-DZVP-rev2 basis sets^51^ and the same *k*-point grid as in the Quantum ESPRESSO calculations. The Raman spectra were computed at the corresponding equilibrium geometries.

## Results

### Single-Crystal XRD Structural Determination

Given the existing controversy about the structure of bismutite (BiO)_2_(CO_3_) at room conditions, we first intend to solve it from our recrystallized sample. The collected diffraction data resulted in approximately 50,000 reflections, likely associated with the high degree of crystalline domains. Of these, around 2000 reflections (∼4.5%) were successfully indexed using an orientation matrix refined with an indexing tolerance parameter of 0.05. The indexed reflections were consistent with an orthorhombic structure in the *Cmcm* space group with lattice parameters *a* = 5.4660(4) Å, *b* = 13.682(5) Å, *c* = 10.9307(8) Å (unit-cell volume of *V* = 817.5(3) Å^3^ and *Z* = 8). While the main diffraction peaks can be indexed using the lattice parameters reported previously, the experimentally determined unit-cell parameters and proposed space group are supported by additional reflections of significant intensity that cannot be accounted for by the reported unit cells (reflection (131), for instance, as shown in Figure S1 of Supporting Information). The initial structure solution allowed determining the positions of the bismuth atoms, the oxygen atoms belonging to the dibismuthyl group and the carbon atoms, based on charge density. These Bi and O atoms adopt, as previously reported, the characteristic (BiO)_2_^2+^ double-layered structure. However, the location of the oxygen atoms belonging to the carbonate groups and, therefore, the orientation of the [CO_3_] units could not be easily determined. The refined bismutite structure contains two independent carbon atoms, C(1) and C(2), in the asymmetric unit. The oxygen atoms bonded to C(1) were positioned from the electron-density map and the C(1)–O bond lengths were restrained to 1.25(5) Å and O–C–O angles to 120°, yielding an intramolecular O–O distance of 2.2(1) Å. For the carbonate group associated with C(2), the oxygen atoms were placed geometrically, as no clear charge density maxima were observed. The same distance and angular restraints were applied. The *U*_eq_ values of the oxygen atoms were constrained to 1.5 times that of their bonded carbon, and the occupancies of the oxygen atoms were constrained and adjusted to maintain the compound’s expected stoichiometry. The best refined structural solution models have multiple orientations of the carbonate groups with O atoms in fractional occupancies, as depicted in Figure 1. The [C(1)­O_3_] and [C(2)­O_3_] carbonate groups have two and four symmetry-equivalent orientations tilted approximately 30° with respect to the (100) plane, respectively. The four [C(2)­O_3_] groups are related pairwise by a 180° rotation around the [100] axis. Details of the XRD data collection and refinement results, the atomic coordinates and the anisotropic displacement parameters obtained for bismutite are provided in Tables 1, S1, and S2 of the Supporting Information, respectively. It should be noted that, although we cannot exclude the possibility that the resolved phase is a metastable one quenched to ambient conditions, its crystal lattice fully accounts for the diffraction peaks observed in the XRD pattern of the original polycrystalline powder, as will be shown below.

**Figure 1:**
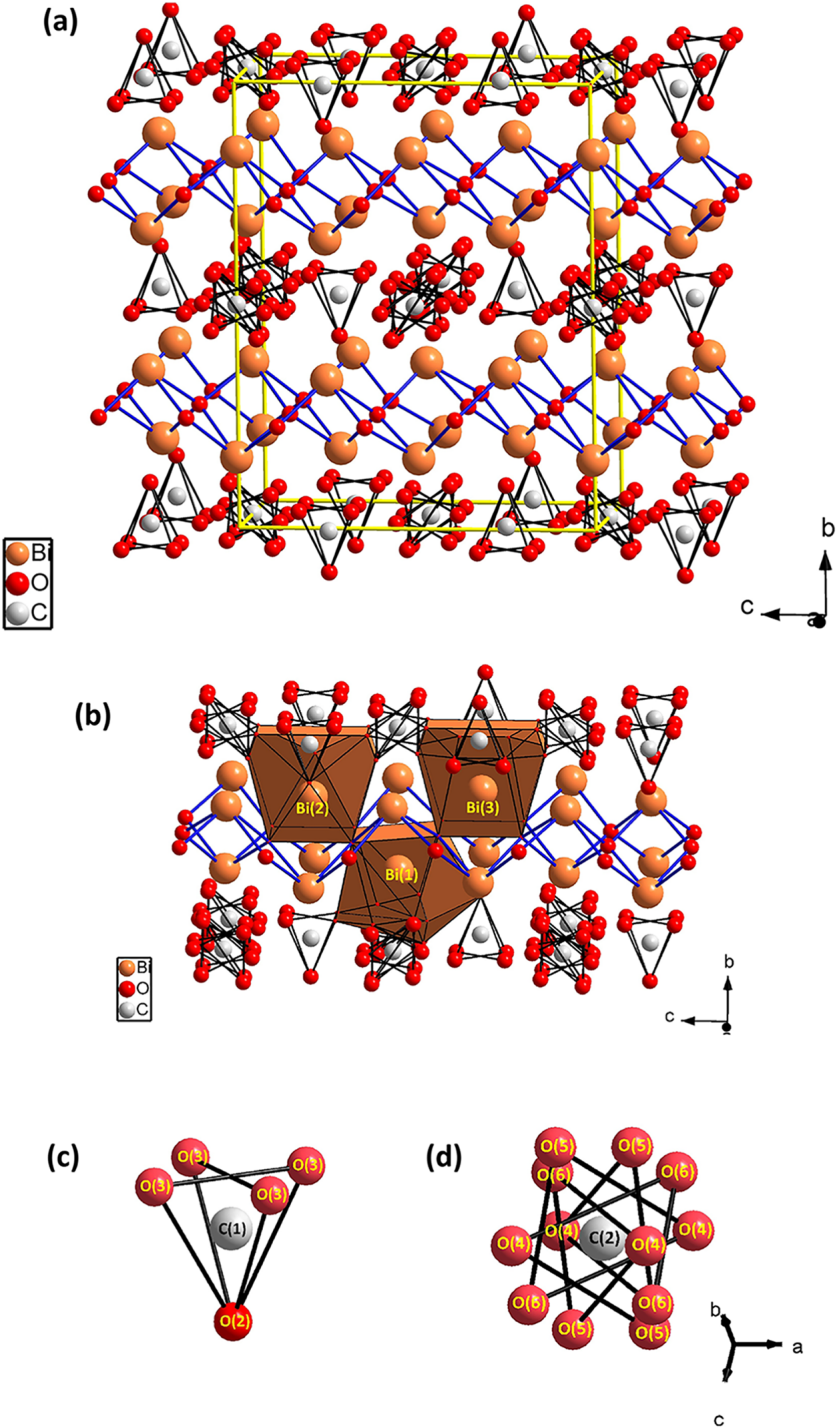
- (a) *Cmcm* (BiO)_2_CO_3_ structure at room conditions. Thick yellow lines indicate the unit-cell. Orange, light gray, and red spheres represent Bi, C, and O atoms, respectively. Blue lines are depicted to illustrate the Bi–O connectivity within the (BiO)_2_^2+^ layers. [CO_3_] trigonal planar units are also represented. (b) (BiO)_2_^2+^ layers in more detail, with three types of Bi-centered polyhedra. (c, d) show the different orientations of [C(1)­O_3_] and [C(2)­O_3_] carbonate groups, respectively.

**Table 1:** Crystal Data, Data Collection, and Structure Refinement Parameters for Bismutite

crystal data	
CCDC number	2506098
empirical formula	Bi_2_CO_5_
formula weight [g mol^–1^]	509.49
crystal system	orthorhombic
space group (number)	*Cmcm* (63)
*a* [Å]	5.4649(3)
*b* [Å]	13.682(4)
*c* [Å]	10.9219(6)
volume [Å^3^]	816.7(3)
*Z*	8
ρ_calc_ [g·cm^–3^]	8.296

data collection	
F(000)	1696
radiation	synchrotron (λ = 0.2904)
2θ range [°]	3.278–27.98
index ranges	–9 ≤ *h* ≤ 9
	–14 ≤ *k* ≤ 14
	–18 ≤ *l* ≤ 18
reflections collected	6223
independent reflections	693

crystal structure refinement	
data/restraints/parameters	693/9/44
goodness-of-fit on *F* ^2^	1.144
final R Indexes [*I* ≥ 2σ(*I*)]	*R*_1_ = 0.0305
	w*R*_2_ = 0.0711
final R indexes [all data]	*R*_1_ = 0.0467
	w*R*_2_ = 0.0808
Δρ_max_/Δρ_min_ [eÅ^–3^]	2.22/-2.80

The (BiO)_2_(CO_3_) structure, shown in Figure 1, can be described as formed by (Bi_2_O_2_)^2+^ layers built from three crystallographically independent bismuth polyhedra: two [Bi­(2,3)­O_10_] bicapped square antiprisms and one [Bi(1)­O_12_] cuboctahedron. The analysis of the coordination was carried out using atomic Voronoi–Dirichlet polyhedral methods^52^ as implemented in the ToposPro software.^53,54^ Each Bi atom is in the +3 oxidation state and possesses a stereochemically active lone electron pair, which strongly influences the topology of the structure as well as the location and orientation of the planar [CO_3_]^2–^ groups. Consequently, the carbonate groups are arranged perpendicular to the (Bi_2_O_2_)^2+^ layers and are oriented such that their oxygen atoms provide sufficient space to accommodate the stereochemically active lone electron pairs.

### DFT Results

We performed DFT calculations of the different experimental structures proposed in the literature, as well as the newly determined structure, to evaluate their stability and how they compare energetically with each other and with our structural determination. In our calculations we considered initially two orthorhombic phases: *Imm*2 (Z = 2)^15^ and *Pna*2_1_ (*Z* = 8).^14^ The third previously experimentally reported structure, with space group *I*4/*mmm*,^12^ is a disordered version of *Imm*2, and therefore we initially considered only the latter in our calculations as a representative of the two. The zero-pressure geometry relaxation of these two structures shows that *Pna*2_1_ is predicted to be significantly more stable than *Imm*2, by about 1 eV per formula unit. Examination of the room-pressure structures reveals that the experimentally reported *Imm*2 phase has O···O contacts between adjacent carbonate groups at a very short distance of 1.65 Å, well below the sum of van der Waals radii for this contact (3.04 Å). After the geometry relaxation, this distance increases to 2.30 Å. Although not as extreme, it is still a short contact which may explain the relative instability of this structure in the DFT calculations.

Figure 2 shows the energy/volume and enthalpy/pressure curves for these calculated phases in the bismutite system. The stability gap between the two phases is clearly shown in both diagrams, and the H­(p) curve shows that the difference in stability increases with pressure, with an enthalpy difference of up to about 2 eV at 30 GPa. We attempted to calculate the Raman spectra associated with the zero-pressure structure of the *Imm*2 phase. The relaxation with the B3LYP functional yields imaginary frequencies at Γ, indicating the structure is not dynamically stable. The atomic motions associated with the imaginary frequencies correspond to carbonate rotations. The dynamical instability of the *Imm*2 phase prompted us to search for an alternative structure based on the disordered *I*4/*mmm* phase. After exploring different placements of the carbonate groups and carrying out geometry relaxations, we arrived at a structure described with the space group *P*4_2_ *nm*. As indicated in Figure 2, this phase is more stable than *Imm*2, but still not competitive with the *Pna*2_1_ phase at any pressure.

**Figure 2:**
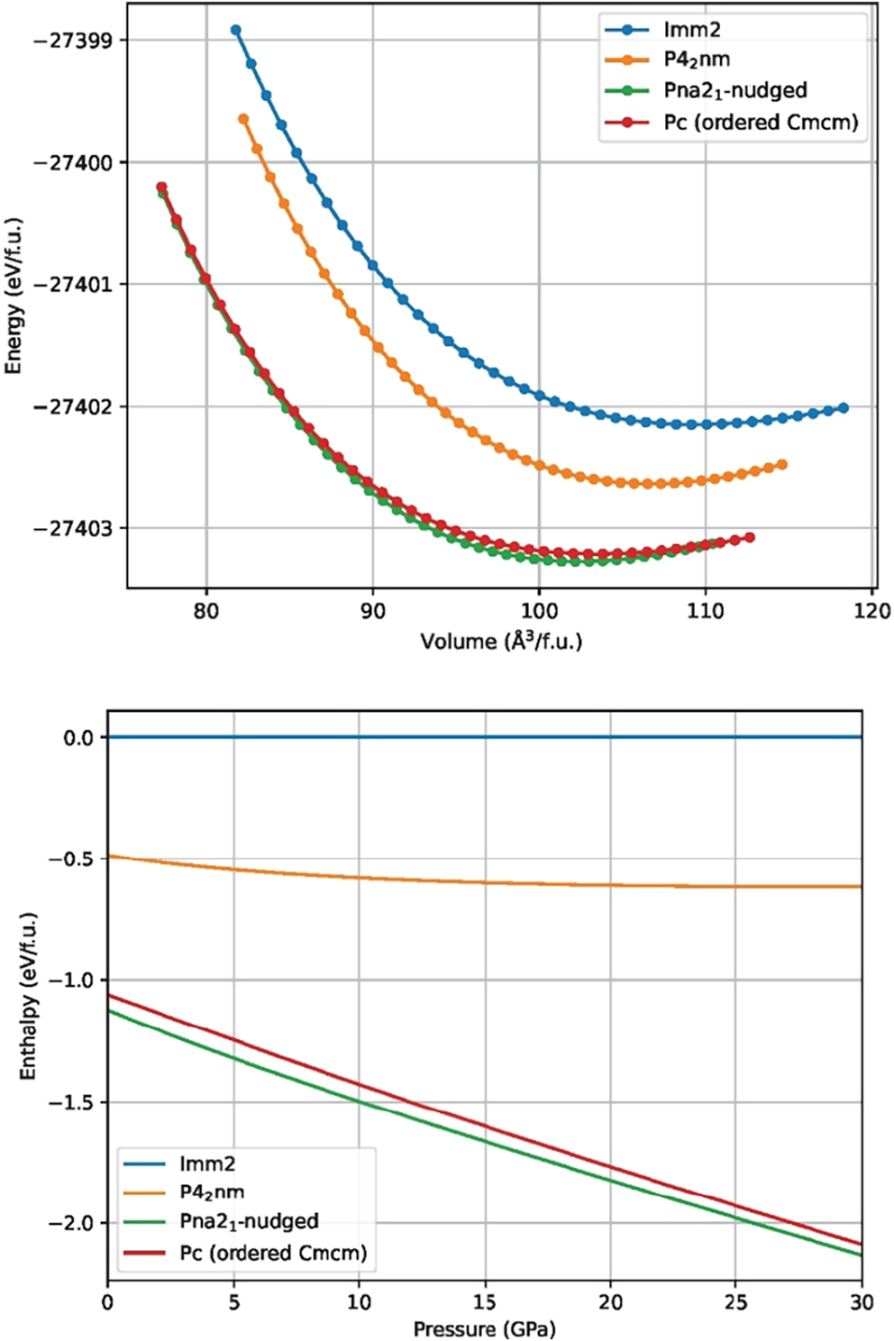
(Top) DFT energy as a function of the volume per (BiO)_2_CO_3_ formula unit for different phases: experimentally reported *Imm*2 ^15^ and *Pna*2_1_,^14^ *P*4_2_ *nm* obtained after geometrical relaxation of alternative [CO_3_] placements within the *Imm*2 lattice dimensions, “*Pna2*_1_-nudge” which is obtained after doubling the *b* axis of the *Pna*2_1_ phase (as suggested the phonon at negative frequency of the latter), and the *Pc* phase derived from relaxation of the experimentally found *Cmcm* structure. (Bottom) Enthalpy difference as a function of pressure, showing the stability of all these structural candidates with respect to *Imm*2 phase of­(BiO)_2_CO_3_ bismutite.

Phonon calculations on the *Pna*2_1_ bismutite phase show that there are imaginary frequencies out of Γ, at (1/2,0,0), indicating that doubling the cell parameter in that direction may result in symmetry breaking and a lower energy (a vibrational band structure plot is shown in Figure S2 of the Supporting Information). We doubled the unit cell in the *a* direction, nudged the structure by displacing it along the eigenvector corresponding to the imaginary frequency phonon, then relaxed the resulting structure. The equilibrium structure obtained (S.G. *Pc*, *a* ∼ 5.47 Å, *b* ∼ 27.60 Å, *c* ∼ 10.84 Å, with α, β and γ angles of 90°), so-called “*Pna*2_1_-nudge”, is considerably more stable than the *Pna*2_1_ phase. This tentative structure shows a slight reorientation of the carbonates in a way that minimizes the interaction between the bismuth lone pairs and the adjacent [CO_3_] planar groups. Further sampling of [CO_3_] orientations did not result in a more stable structure.

Additional calculations were carried out on the experimentally solved *Cmcm* structure assuming an ordered arrangement of the carbonate oxygen atoms, leading to a reduction in symmetry to the *Pc* (S.G. Nr. 7) description (*a*_*T*_ = 13.835 Å, *b*_*T*_ = 10.877 Å, *c*_*T*_ = 5.474 Å, β_*T*_ = 91.6°, at 0.23 GPa). These calculated lattice parameters correspond to the experimental *b, c* and *a* axes, respectively. The atomic coordinates of this DFT-calculated *Pc* structure are collected in Table S3 of the Supporting Information. This structure is dynamically stable, and its enthalpy is similar to that of the “*Pna*2_1_-nudge” structure we obtained by following the imaginary-frequency phonon eigenvectors of the original *Pna2*_*1*_ structure. Both structures were, in any case, significantly more energetically stable than the *Imm*2 structure.

To evaluate whether the experimental data favored an ordered monoclinic model over the disordered orthorhombic description, the experimental data set was intentionally indexed and tested in a monoclinic setting. The resulting experimental monoclinic unit cell parameters were *a* = 5.4655(3) Å, *b* = 13.679(6) Å, *c* = 10.9239(7) Å, and β = 90.05°. The metric symmetry of this experimentally derived unit cell clearly indicates a predominantly orthorhombic character. Furthermore, successful structure solution was not possible using the DFT-calculated *Pc* setting, supporting the preference for the experimentally determined disordered *Cmcm* model. Lastly, the “*Pna*2_1_-nudge” structure was one among several phases we studied that were obtained by nudging the original *Pna*2_1_ structure. All these structures, and the *Pc* phase, are similar in enthalpy within the studied pressure range, suggesting the existence of dynamic disorder.

### Structural Evolution upon Compression

The structural evolution of bismutite, (BiO)_2_(CO_3_), at high pressure and room temperature has been studied by means of *in situ* powder synchrotron X-ray diffraction measurements. The experiments were performed under hydrostatic conditions using Ne as pressure transmitting medium. Figure 3 illustrates a selection of patterns at different pressures which shows that, up to 17.7 GPa, there are no obvious changes in the XRD profiles except for the shift of the peaks to higher 2θ angles as a result of unit cell compression. The fact that the high-pressure synchrotron powder XRD patterns present some texturing effects due to uneven crystal sizes, together with weakly scattering oxygen and carbon atoms compared with heavy bismuth atoms, precludes full structural Rietveld refinements. Therefore, only LeBail refinements were performed.

**Figure 3:**
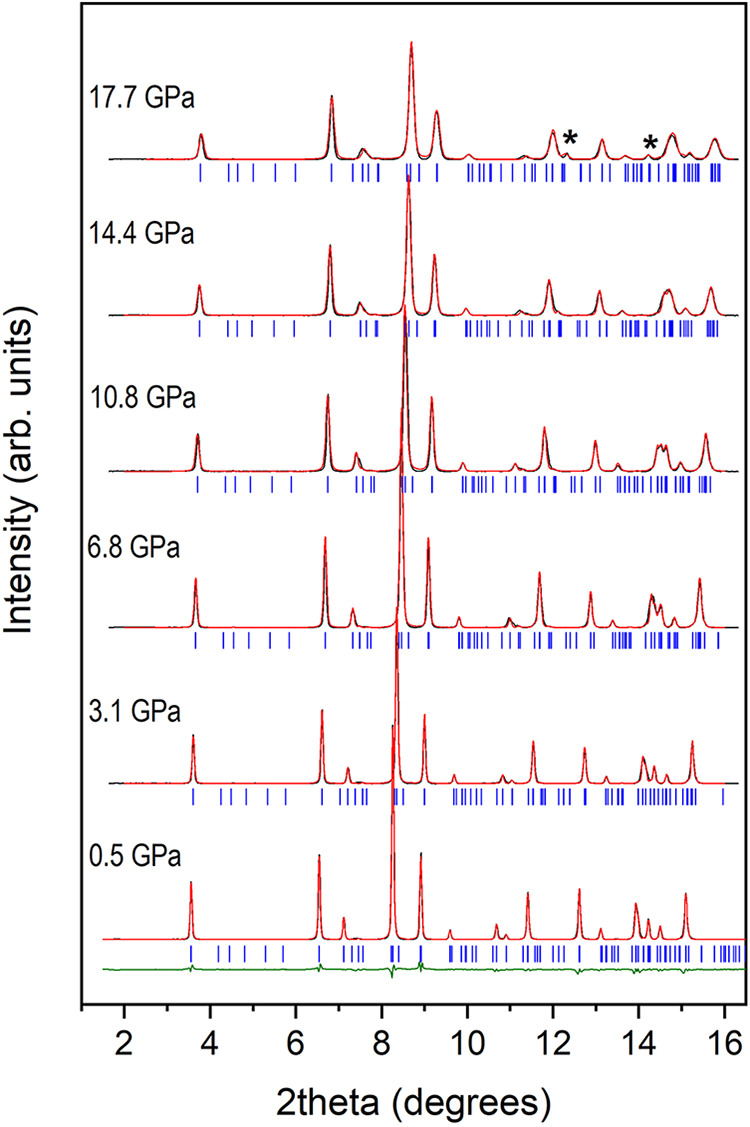
- Selected powder XRD patterns of bismutite up to 17.7 GPa (λ = 0.4246 Å). All the patterns can be indexed with an orthorhombic *Cmcm* unitcell and no significant changes in relative peak intensities changes are observed in the studied pressure range, which suggest the absence of phase transitions. The diffraction signals corresponding to the neon pressure-transmitting medium are marked with asterisks. Backgrounds have been subtracted. The red solid lines correspond to the Le Bail fitprofiles, while blue sticks indicate the Bragg positions for the *Cmcm* phase.

All the high-pressure XRD patterns up to 17.7 GPa can be indexed using an orthorhombic *Cmcm* unit cell. The experimental lattice parameters and unit cell volumes at different pressures are collected in Table 2 and their evolution as a function of pressure is represented in Figures 4 and 5, respectively. As can be seen, the lattice parameters vary smoothly with increasing pressure, in reasonable agreement with theory (also depicted in Figures 4 and 5), which supports the absence of first-order phase transitions in this pressure range. It is noteworthy that the lattice parameters upon decompression precisely retrace those observed during compression, thereby accounting for the single-crystal synthesis process. The main difference between the theoretical (T subscript hereafter) and experimental data is that the calculations suggest a smaller compressibility for the calculated *b*_*T*_ lattice parameter associated with the experimental *c* axis. This discrepancy may be partly attributable to the arbitrary choice of the carbonate group positions. The monoclinic β angle of the calculated structure decreases with pressure (see inset of Figure 4).

**Figure 4:**
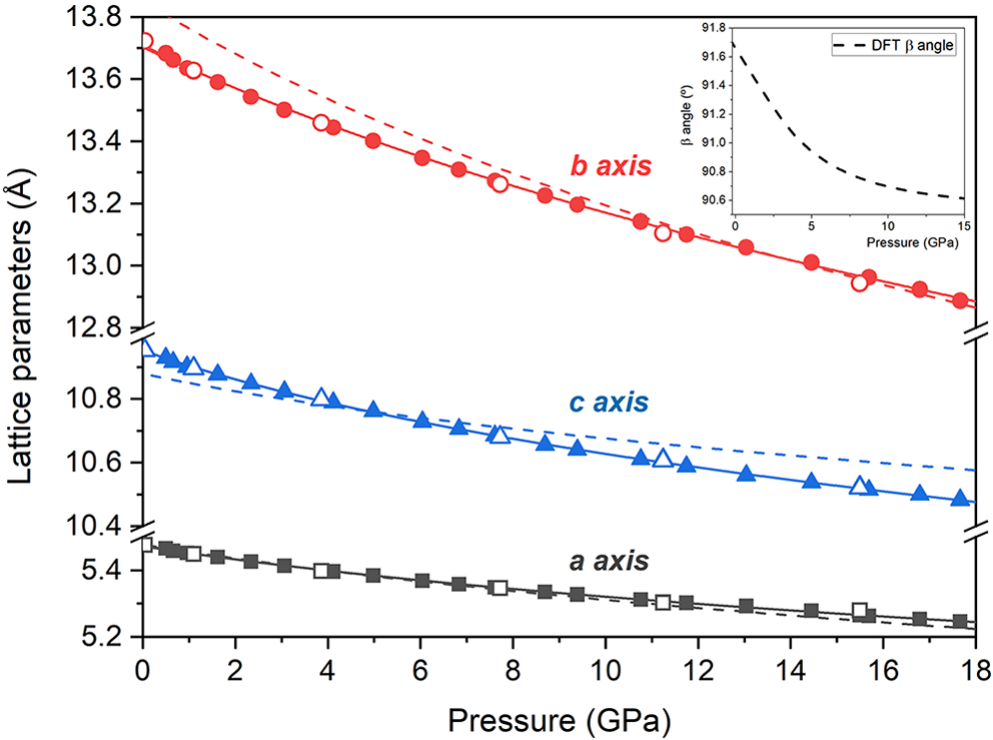
Pressure dependence of the *Cmcm* (BiO)_2_CO_3_ lattice parameters up to 17.7 GPa. Black squares, red circles and blue triangles represent the experimental *a*, *b* and *c* axes, respectively. Solid and empty symbols correspond to the upstroke and the downstroke, respectively. Solid lines represent the 3rd-order BM axial fits, while the dashed lines are results from DFT calculations. Inset: Variation of DFT-calculated monoclinic *Pc* β angle with increasing pressure. The estimated standard deviation values in the experimental data are smaller than the dimensions of the symbols.

**Figure 5:**
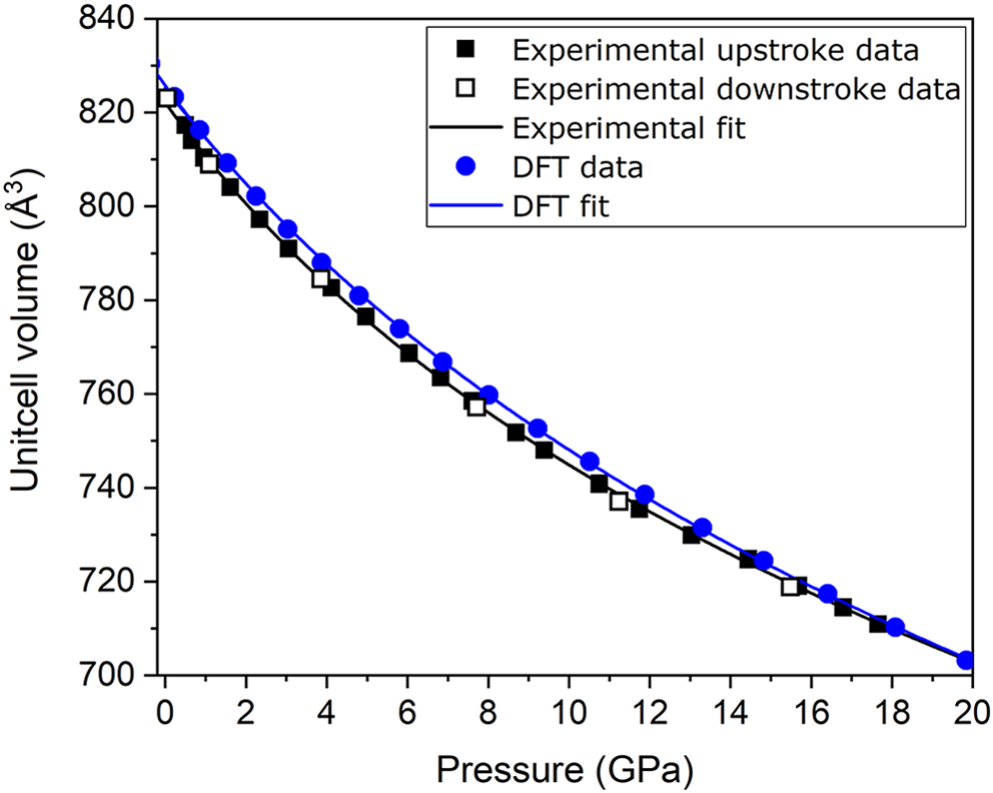
Unit-cell volume vs pressure for (BiO)_2_CO_3_ bismutite. Black squares and blue circles represent experimental and DFT data, respectively, and the solid lines lines the fitted equation of state. Solid and empty symbols correspond to upstroke and downstroke data, respectively. The estimated standard deviation values in the experimental data are smaller than the dimensions of the symbols.

**Table 2:** Experimental Unit Cell Parameters and Volumes of Cmcm (BiO)_2_CO_3_ at Different Pressures^a^

pressure (GPa)	*a* axis (Å)	*b* axis (Å)	*c* axis (Å)	unit cell volume (Å^3^)
0.50(5)	5.465(2)	13.682(5)	10.929(4)	817.2(4)
0.66(5)	5.458(2)	13.662(5)	10.916(4)	814.0(4)
0.96(5)	5.452(2)	13.635(5)	10.902(4)	810.4(4)
1.62(5)	5.439(2)	13.590(5)	10.877(4)	804.0(3)
2.34(5)	5.426(2)	13.543(5)	10.849(4)	797.2(3)
3.06(5)	5.413(2)	13.500(5)	10.823(4)	790.9(3)
4.12(5)	5.396(2)	13.444(5)	10.789(4)	782.7(3)
4.98(5)	5.384(2)	13.402(5)	10.762(4)	776.5(3)
6.04(5)	5.368(2)	13.347(5)	10.729(4)	768.7(3)
6.83(5)	5.358(2)	13.309(5)	10.706(4)	763.4(3)
7.61(5)	5.348(2)	13.273(5)	10.685(4)	758.5(3)
8.69(5)	5.335(2)	13.226(5)	10.656(4)	751.9(3)
9.39(5)	5.327(2)	13.196(5)	10.640(4)	748.0(3)
10.76(5)	5.312(2)	13.143(5)	10.611(4)	740.9(3)
11.75(5)	5.302(2)	13.101(5)	10.588(4)	735.4(3)
13.04(5)	5.293(2)	13.059(5)	10.561(4)	729.9(3)
14.45(5)	5.279(2)	13.011(5)	10.537(4)	723.7(3)
15.7(1)	5.263(2)	12.962(5)	10.514(4)	717.3(3)
16.8(1)	5.253(2)	12.922(5)	10.499(4)	712.7(3)
17.7(1)	5.246(2)	12.888(5)	10.484(4)	708.8(3)
15.5(1) – D*	5.265(2)	12.942(5)	10.522(4)	717.0(3)
11.24(5) – D*	5.303(2)	13.103(5)	10.608(4)	737.1(3)
7.72(5) – D*	5.346(2)	13.261(5)	10.680(4)	757.2(3)
3.86(5) – D*	5.398(2)	13.459(5)	10.799(4)	784.5(3)
1.10(5) – D*	5.448(2)	13.626(5)	10.896(4)	808.9(3)
0.05(5) – D*	5.476(2)	13.723(5)	10.952(4)	823.0(3)

a D* indicates measurements during decompression.

The DFT calculated volume (monoclinic *Pc* space group) is overestimated by approximately 0.5% with respect to the experimental value at ambient conditions, as a consequence of a slight overestimation of the *b*_*T*_ axis length, which corresponds to the experimental *c* axis. The pressure evolution of the experimental lattice parameters (Figure 4) shows that the *b* axis (*a*_*T*_) is the more compressible. A linearization of the third-order Birch–Murnaghan equation of state (through the substitution of the volume for the cube of a lattice parameter) was used to analyze the axial compression,^55^ providing evidence of the anisotropy in this compound. Thus, the experimental axial bulk moduli values obtained from data up to 17.7 GPa are B_*a*_ = 83(3) GPa (B′_0a_ = 8.7(5)) and B_*c*_ = 70.4(17) GPa (B′_0c_ = 10.7(4)), for the *a* and *c* axes, respectively, which represents a large variation of the compressibility with increasing pressure. The *b* axis, on the other hand, has a larger compressibility (B_*b*_ = 64.6(12) GPa and B′_0b_ = 4.4(2)). These experimental values of the axial bulk moduli and their first-pressure derivatives are in good agreement with the calculated data: B_*aT*_ = 49.8(7) GPa (B′_0*aT*_ = 4.39(9)), B_*bT*_ = 115(2) GPa (B′_0*bT*_ = 16.5(5)) and B_*cT*_ = 76.4(9) GPa (B′_0*cT*_ = 7.6(2)), as depicted in Figure 4. The larger decrease in compressibility as a response to external pressure along the *a* and *c* axes arises from the orientation of the highly incompressible [CO_3_] carbonate units with respect to the region where the lone electron pairs of the Bi atoms are (see Figure 2).

The evolution of the unit cell volume as a function of pressure can be explained with a third-order Birch–Murnaghan equation of state (BM-EoS)^55^ with a zero-pressure volume V_0_ = 822.2(4) Å^3^, a bulk modulus of B_0_ = 67.2(11) GPa, and a bulk modulus first-pressure derivative of B_0_′ = 8.6(2). These experimental results compare well with those obtained from our DFT calculations: V_0_ = 825.3(4) Å^3^, B_0_ = 73.5(11) GPa, and B_0_′ = 6.74(19). The bulk modulus of (BiO)_2_(CO_3_) bismutite is (i) larger than those reported for other carbonates of monovalent cations like Ag_2_CO_3_ (B_0_ = 42.9 GPa, B_0_′ = 7.2)^56^ or the γ-phase of Na_2_CO_3_ (B_0_ = 38.1 GPa, B_0_′ = 5.2),^57^ (ii) slightly larger than those of carbonates with large divalent cations like Sr, Ba or Pb (45 < B_0_ < 67 GPa),^58–61^ and (iii) smaller than those of carbonates of divalent cations, which range between 131 and 67 GPa.^62–64^ Bismutite’s bulk modulus can be explained by the fact that its structure is an intergrowth of (BiO)_2_^2+^ layers, similar to those existing in α-Bi_2_O_3_, and highly incompressible [CO_3_]^2–^ connecting anions. Note that the bulk modulus of the α polymorph of bismuth oxide (90–106 GPa)^65^ is larger than that of the carbonate and, therefore, the compressibility of the latter could be understood only in terms of a more efficient accommodation between the carbonate groups and the bismuth lone electron pairs.

To further understand the compressibility of the different crystallographic axes, we calculated the evolution of the lone electron pair (LEP) volume as a function of pressure. In our calculations, the LEPs are represented by the basins of the electron localization function.^66^ These volumes were obtained by integration of the CRYSTAL17 electron densities calculated on a grid using the Yu-Trinkle algorithm^67^ implemented in critic2.^68^ Figure S3 shows the DFT-calculated *Pc* unitcell and the location of the ELF maximum associated with the LEP outlined in red. At 0.23 GPa, the LEP volume is 8.39 Å^3^ and the location of the LEP is 1.24 Å away from the position of its Bi atom. The compressibility of this ELF basin (B_0_ = 38.8(7) GPa, B_0_′ = 2.78(10)) is considerably larger than the bulk compressibility of bismutite. This fact, together with the orientation of the LEP along the [1 0 0]_T_ direction, explains the more marked decrease of the *a*_T_ lattice parameter (the experimental *b* axis) with increasing pressure.

### Vibrational Behavior at High Pressures from Raman Spectroscopy

Raman spectroscopy is a versatile tool for studying the properties of carbonate minerals^69^ and a very sensitive technique to identify phase transitions upon compression.^70^ The Raman spectrum recorded under ambient conditions is shown in Figure 6. The overall appearance of the spectrum is similar to those reported elsewhere.^71,72^ Group theoretical analyses predict 78 Raman active modes for the *Cmcm* structure: Γ_R_ = 21A_g_ + 20B_1g_ + 16B_2g_ + 21B_3g_. It is not possible to identify such a large number of bands in the experimental spectrum, which is dominated by broad bands. However, the DFT simulation based on the *Pc* phase shows a qualitative agreement with the measured spectrum. We will discuss here the characteristics of the spectrum related to the carbonate groups in order to extract information about their configuration.

**Figure 6:**
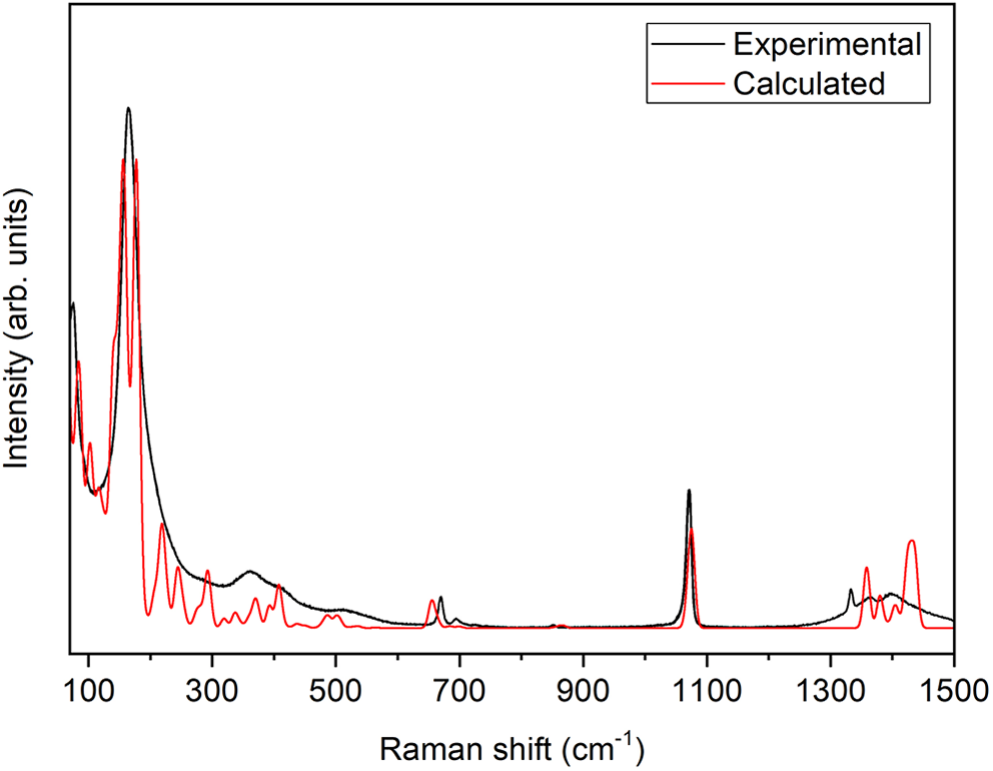
Comparison between calculated (red line, *Pc*) and experimental (black line, *Cmcm*) Raman spectra of the (BiO)_2_CO_3_ bismutite at room pressure. Calculated Raman spectra were used for the mode assignment of Raman frequencies of the experiment.

In general, the spectra of carbonates can be divided into two regions.^73^ Those bands at frequencies above 600 cm^–1^ are mainly due to the internal motions of the molecular carbonate ion. Those below 600 cm^–1^ are lattice modes. Our spectrum clearly shows this distinction. In order to interpret the spectrum, we provide further details about the carbonate ion vibrations. An isolated carbonate ion would have D_3h_ point-group symmetry. The molecule vibrations can be described as Γ_molec_=*A*_1_^^′^^+*A*_2_^^″^^+2*E* ^′^. The one-dimensional *A*_1_^^′^^ vibration, usually labeled *v*_1_, corresponds to symmetric stretching and is expected to be observed around 1088 cm^–1^. The *A*_2_^^″^^ (*v*_2_) out-of-plane bending is close to 838 cm^–1^. The remaining *E* ^′^ vibrations are 2-fold degenerated. The one with largest wavenumber (*v*_3_, 1607 cm^–1^) is associated with the asymmetric stretching, while the other one (*v*_4_, 655 cm^–1^) is related to in-plane bending. All modes are Raman active, except *A*_2_^^″^^ (*v*_2_), which is only IR active. The experimental spectrum shows modes close to the anticipated wavenumbers but with unexpected multiplicities. In particular, the region between 1330 and 1440 cm^–1^ displays four bands. This fact can only be explained if not only the degeneracy of the *v*_3_ (*E* ^′^) mode is broken, but also if carbonate ions are situated at two different crystallographic positions. According to subduction tables, *E* ^′^ degeneracy is only broken if the three-order axis is lost, reducing symmetry to at least *C*_2*v*_. The description of the bismutite structure based on the *Cmcm* spatial group includes two different crystallographic sites for carbon without three-order symmetry. It is thus compatible with the *v*_3_ splitting. The *v*_2_ vibration in a carbonate ion without a three-order axis would become Raman active. A very weak mode is indeed observed at 850.9 cm^–1^. This picture, however, faces two difficulties. First, in the region between 669.8 and 700 cm^–1^, corresponding to *v*_4_ (*E* ^′^), we expect four modes, whereas only three clear features are observed. Second, the *v*_1_ (*A*_1_^^′^^) phonon should become a doublet, but at 1071.5 cm^–1^ we observe a single mode, whose asymmetry is not conclusive.

To address these difficulties, Raman scattering spectra at some representative pressures were taken; they are depicted in Figures 7 and S4, and the evolution of the Raman modes frequencies with increasing pressure, in Figure 8. We observe a progressive widening of the *v*_1_ (*A*_1_^^′^^) band which, above 14 GPa, is clearly split into two well-defined Raman bands corresponding to the two crystallographically distinct carbon atoms. Carbonate ions are oriented perpendicular to polymeric (Bi_2_O_2_)^2+^ layers (Figure 1). As pressure increases and interlayer space is reduced, we expect that both the symmetry reduction and differences in carbon crystal sites to become more apparent. The symmetry effect explains why the intensity of the *v*_2_ increases as pressure is applied. However, site symmetry seems to not induce an evident doubling of this mode. It should be recalled that this mode is related to out-of-plane bending. The movement of atoms in the direction parallel to polymeric (Bi_2_O_2_)^2+^ layers is expected to be less affected by interlayer compression. In fact, the *v*_2_ frequency remains nearly constant under high pressure. Finally, the splitting of the *v*_4_ mode into four bands is also better appreciated under high pressure (see spectrum at 8.4 GPa in Figure S5).

**Figure 7:**
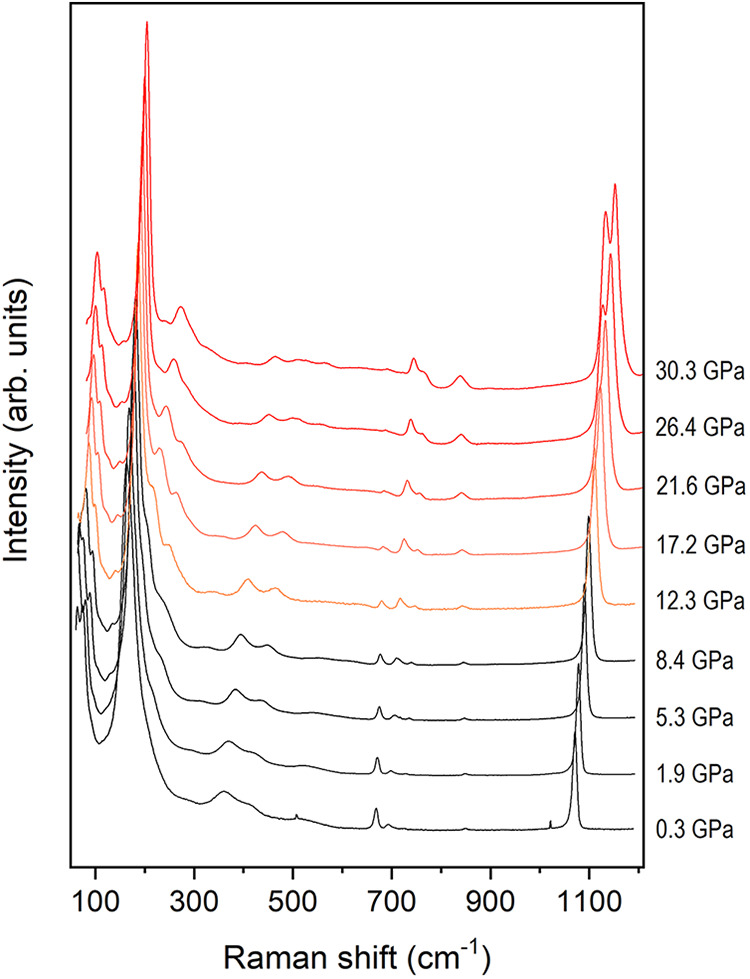
Room-temperature Raman spectra of (BiO)_2_CO_3_ bismutite at selected pressures on upstroke in the frequency region 60–1200 cm^–1^. The spectra in a reddish color correspond to the pressure range where progressive changes in the relative intensity of certain bands and a band splitting in the [CO_3_] stretching frequency region appear.

**Figure 8:**
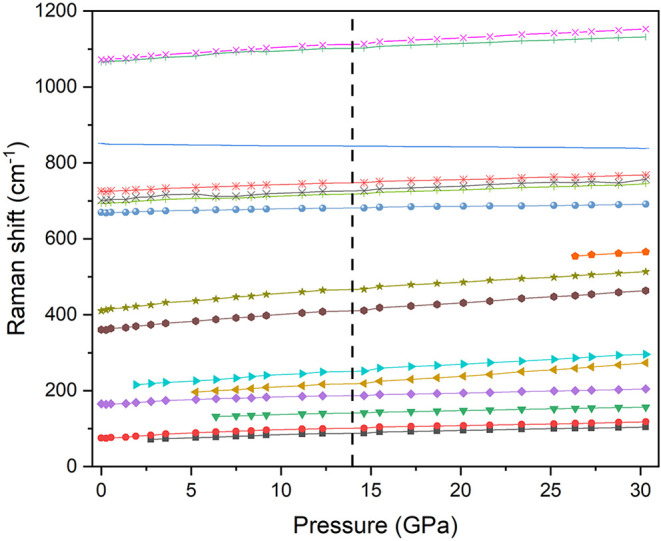
Pressure evolution of the Raman modes of (BiO)_2_CO_3_. Solid symbols represent the experimental data obtained upon compression. Solid lines represent the evolution of the experimentally observed modes. The dashed line marks the pressure at which a clear differentiated behavior of the two nonequivalent carbon atoms is observed.

## Conclusions

In this work we carried out a joint experimental/computational investigation of the structure and behavior of (BiO)_2_(CO_3_) bismutite at ambient conditions and under high pressure. Single-crystal X-ray diffraction analysis reveals that (BiO)_2_(CO_3_) bismutite crystallizes in the orthorhombic space group *Cmcm*. Its structure comprises (BiO)_2_^2+^ layers interleaved with [CO_3_]^2–^ moieties that exhibit structural disorder at ambient conditions, consistent with that suggested in previous reports. The orientation of these [CO_3_] units was unambiguously determined and is governed by the lone-pair distribution of the Bi atoms.

In situ structural characterization under high-pressure conditions reveals the compressibility behavior of this material and its inherent anisotropy. These results are in excellent agreement with density-functional theory calculations, which indicate that the high compressibility of the ELF basin associated with the Bi atom lone electron pair is an important factor governing the decrease of the interlayer distance and the overall bulk compressibility of bismutite. Above 14 GPa, Raman spectroscopy results evidence clearly differentiated behaviors for the two crystallographically independent carbon atoms. Specifically, the spectra exhibit a distinct splitting that can only be accounted for if inequivalent local environments affecting the force constants of the carbonate groups occur.

This work sheds light on the long-standing controversy regarding the structure of (BiO)_2_(CO_3_) bismutite. Furthermore, it reports for the first time the behavior of this compound, which belongs to the Aurivillius and Sillen phases’ family, under compression, and reveals the role of the lone electron pairs associated with the Bi atom and the orientation of the [CO_3_] carbonate groups in determining its final topology.

## Supplementary Material



## Notes

Tables with experimental XRD and theoretical DFT results and structural details. Additional figures with data analyses (PDF)

## References

[1] Sahama T. G., Lehtinen M. (1968). Bismutite of the granite
pegmatites of Zambezia, Mozambique. Bull. Geol.
Soc. Finland.

[2] Stoltzfus M. W., Woodward P. M., Seshadri R., Klepeis J.-H., Bursten B. (2007). Structure
and Bonding in SnWO_4_, PbWO_4_, and BiVO_4_: Lone Pairs vs Inert Pair. Inorg. Chem..

[3] Pereira A. L. J., Gracia L., Santamaria-Perez D., Vilaplana R., Manjon F. J., Errandonea D., Nalin M., Beltran A. (2012). Structural
and vibrational study of cubic Sb_2_O_3_ under high
pressure. Phys. Rev. B.

[4] Santamaría-Pérez D., Chulia-Jordan R., Rodriguez-Hernandez P., Muñoz A. (2015). Crystal behavior
of potassium bromate under compression. Acta
Crystallogr., Sect. B:Struct. Sci., Cryst. Eng. Mater..

[5] Sans J. A., Manjon F. J., Popescu C., Cuenca-Gotor V. P., Gomis O., Muñoz A., Rodriguez-Hernandez P., Contreras-Garcia J., Pellicer-Porres J., Pereira A. L. J., Santamaria-Perez D., Segura A. (2016). Ordered helium trapping
and bonding in compressed arsenolite:
Synthesis of As_4_O_6_·2He. Phys. Rev. B.

[6] Sidgwick N. V., Powell H. M. (1940). Bakerian Lecture: Stereochemical
types and valency
groups. Proc. R. Soc. London, Ser. A.

[7] Gillespie R. J. (2008). Fifty years
of the VSEPR model. Coord. Chem. Rev..

[8] Bader R. F. W., Gillespie R. J., MacDougall P. J. (1988). A physical basis for the VSEPR model
of molecular geometry. J. Am. Chem. Soc..

[9] Aurivillius B. (1949). Mixed bismuth
oxides with layer lattices I. The structure type of CaNb_2_Bi_2_O_9_. Ark. Kemi.

[10] Sillén L. G. (1942). Über
eine Familie von Oxyhalogeniden. Naturwissenschaften.

[11] Levitskaia T. G., Qafoku N. P., Bowden M. E., Asmussen R. M., Buck E. C., Freedman V. L., Pearce C. I. (2022). A Review
of Bismuth­(III)-Based Materials
for Remediation of Contaminated Sites. ACS Earth
Space Chem..

[12] Lagercrantz A., Sillen L. G. (1948). On the crystal structure of Bi_2_O_2_CO_3_ (bismutite) and CaBi_2_O_2_(CO_3_)_2_ (beyerite). rk. Kemi,
Mineral. Geol. A.

[13] Bhachu D. S., Moniz S. J. A., Sathasivam S., Scanlon D. O., Walsh A., Bawaked S. M., Mokhtar M., Obaid A. Y., Parkin I. P., Tang J., Carmalt C. J. (2016). Bismuth oxyhalides: synthesis, structure
and photoelectrochemical activity. Chem. Sci..

[14] Greaves C., Blower S. K. (1988). Structural relationships
between Bi_2_O_2_CO_3_ and β-Bi_2_O_3_. Mater. Res. Bull..

[15] Grice J. D. (2002). A solution
to the crystal structures of Bismutite and Beyerite. Can. Mineral..

[16] Huang H., Tian N., Jin S., Zhang Y., Wang S. (2014). Syntheses,
characterization and nonlinear optical properties of bismuth subcarbonate
Bi_2_O_2_CO_3_. Solid
State Sci..

[17] Henmi H., Hirayama T., Sawada Y., Mizutani N., Kato M. (1987). Thermal decomposition
of basic bismuth carbonate under high pressures of CO_2_ and
N_2_ to 50 atm. Thermochim. Acta.

[18] Sheng S., Jin S., Cui K. (2020). Thermal decomposition
of nanostructured bismuth subcarbonate. Materials.

[19] Santamaria-Perez D., Otero-de-la-Roza A., Ruiz-Fuertes J., Chulia-Jordan R., Marqueño T., MacLeod S., Popescu C. (2020). Pressure and temperature
effects on low-density Mg_3_Ca­(CO_3_)_4_ huntite carbonate. J. Phys. Chem. C.

[20] Pereira A. L. J., Errandonea D., Beltrán A., Gracia L., Gomis O., Sans J. A., García-Domene B., Miquel-Veyrat A., Manjón F. J., Muñoz A. (2013). Structural study of α-Bi_2_O_3_ under pressure. J. Phys.:
Condens. Matter.

[21] Gomis O., Santamaria-Perez D., Ruiz-Fuertes J., Sans J. A., Vilaplana R., Ortiz H. M., García-Domene B., Manjón F. J., Errandonea D., Rodríguez-Hernández P., Muñoz A., Mollar M. (2014). High-pressure structural and elastic
properties of Tl_2_O_3_. J.
Appl. Phys..

[22] Liermann H.-P., Konôpková Z., Morgenroth W., Glazyrin K., Bednarčik J., McBride E. E., Petitgirard S., Delitz J. T., Wendt M., Bican Y., Ehnes A., Schwark I., Rothkirch A., Tischer M., Heuer J., Schulte-Schrepping H., Kracht T., Franz H. (2015). The Extreme Conditions
Beamline P02.2 and the Extreme Conditions Science Infrastructure at
PETRA III. J. Synchrotron Radiat..

[23] Prescher C., Prakapenka V. B. (2015). DIOPTAS:
A program for reduction of two-dimensional
X-ray diffraction data and data exploration. High Pressure Res..

[24] Rigaku Oxford Diffraction. CrysAlisPro Software System. 2024.

[25] Aslandukov A., Aslandukov M., Dubrovinskaia N., Dubrovinsky L. (2022). *Domain
Auto Finder* (*DAFi*) Program: The Analysis
of Single-Crystal X-Ray Diffraction Data from Polycrystalline Samples. J. Appl. Crystallogr..

[26] Dolomanov O. V., Bourhis L. J., Gildea R. J., Howard J. A. K., Puschmann H. (2009). *OLEX2*: A Complete Structure Solution,
Refinement and Analysis Program. J. Appl. Crystallogr..

[27] Sheldrick G. M. (2015). *SHELXT* –
Integrated Space-Group and Crystal-Structure
Determination. Acta Crystallogr., Sect. A:Found.
Adv..

[28] Sheldrick G. M. (2015). Crystal
Structure Refinement with *SHELXL*. Acta Crystallogr., Sect. C:Struct. Chem..

[29] Patterson A. L. (1939). The Scherrer
Formula for X-Ray Particle Size Determination. Phys. Rev..

[30] Hemley R. J., Zha C. S., Jephcoat A. P., Mao H. K., Finger L. W., Cox D. E. (1989). X-Ray diffraction and equation of state of solid neon
to 110 GPa. Phys. Rev. B.

[31] Santamaría-Pérez D., Mukherjee G. D., Schwager B., Boehler R. (2010). High-pressure melting
curve of helium and neon: Deviations from corresponding states theory. Phys. Rev. B.

[32] Mao H. K., Xu J., Bell P. M. (1986). Calibration
of the ruby pressure gauge to 800-Kbar
under quasi-hydrostatic conditions. J. Geophys.
Res..

[33] Fauth F., Peral I., Popescu C., Knapp M. (2013). The new material science
powder diffraction beamline at ALBA synchrotron. Powder Diffr..

[34] Holland T. J. B., Redfern S. A. T. (1997). Unit cell refinement from powder
diffraction data: the use of regression diagnostics. Mineral. Mag..

[35] Nolze G., Kraus W. (1998). Powdercell 2.0 for
Windows. Powder Diffr..

[36] Rodríguez-Carvajal J. (1993). Recent advances
in magnetic structure determination by neutron powder diffraction. Phys. B.

[37] Bayarjargal L., Fruhner C. J., Schrodt N., Winkler B. (2018). CaCO_3_ phase
diagram studied with Raman spectroscopy at pressures up to 50 GPa
and high temperatures and DFT modeling. Phys.
Earth Planet. Inter..

[38] Botan-Neto B. D., Santamaria-Perez D., Bayarjargal L., Bykova E., Gonzalez-Platas J., Otero-de-la-Roza A. (2024). Dense hydrated
magnesium carbonate MgCO_3_·3H_2_O phases. Inorg. Chem..

[39] Blöchl P. E. (1994). Projector
augmented-wave method. Phys. Rev. B.

[40] Giannozzi P., Andreussi O., Brumme T., Bunau O., Buongiorno
Nardelli M., Calandra M., Car C., Cavazzoni C., Ceresoli D., Cococcioni M., Colonna N., Carnimeo I., Dal Corso A., de Gironcoli S., Delugas P., DiStasio R. A., Ferretti A., Floris A., Fratesi G., Fugallo G., Gebauer R., Gerstmann U., Giustino F., Gorni T., Jia J., Kawamura M., Ko H.-Y., Kokalj A., Küçükbenli E., Lazzeri M., Marsili M., Marzari N., Mauri F., Nguyen N. L., Nguyen H. V., Otero-de-la-Roza A., Paulatto L., Poncé S., Rocca D., Sabatini R., Santra B., Schlipf M., Seitsonen A. P., Smogunov A., Timrov I., Thonhauser T., Umari P., Vast N., Wu X., Baroni S. (2017). Advanced capabilities
for materials modelling with Quantum ESPRESSO. J. Phys.: Condens. Matter.

[41] Perdew J. P., Ruzsinzky A., Csonka G. I., Vydrov O. A., Scuseia G. E., Constantin L. A., Zhou X., Burke K. (2008). Restoring
the density-gradient
expansion for exchange in solids and surfaces. Phys. Rev. Lett..

[42] Dal
Corso A. (2014). Pseudopotentials periodic table: From H to Pu. Comput. Mater. Sci..

[43] Otero-De-La-Roza A., Luaña V. (2011). Gibbs2: A
new version of the quasi-harmonic model code.
I. Robust treatment of the static data. Comput.
Phys. Commun..

[44] Otero-De-La-Roza A., Abbasi-Pérez D., Luaña V. (2011). Gibbs2: A
new version of the quasiharmonic
model code. II. Models for solid-state thermodynamics, features and
implementation. Comput. Phys. Commun..

[45] Baroni S., De Gironcoli S., Corso A. D., Giannozi P. (2001). Phonons and related
crystal properties from density-functional perturbation theory. Rev. Mod. Phys..

[46] Maschio L., Kirtman B., Rérat M., Orlando R., Dovesi R. (2013). Ab initio
analytical Raman intensities for periodic systems through a coupled
perturbed Hartree-Fock/Kohn-Sham method in an atomic orbital basis.
I. Theory. J. Chem. Phys..

[47] Maschio L., Kirtman B., Rérat M., Orlando R., Dovesi R. (2013). Ab initio
analytical Raman intensities for periodic systems through a coupled
perturbed Hartree-Fock/Kohn-Sham method in an atomic orbital basis.
II. Validation and comparison with experiments. J. Chem. Phys..

[48] Dovesi R., Erba A., Orlando R., Zicovich-Wilson C. M., Civalleri B., Maschio L. M., Rérat M., Casassa S., Baima J., Salustro S., Kirtman B. (2018). Quantum-mechanical
condensed matter simulations with CRYSTAL. WIREs
Comput. Mol. Sci..

[49] Becke A. D. (1993). Density-functional
thermochemistry. III. The role of exact exchange. J. Chem. Phys..

[50] Lee Ch., Yang W., Parr R. G. (1988). Development of the Colle-Salvetti
correlation-energy formula into a functional of the electron density. Phys. Rev. B.

[51] Oliveira D. V., Laun J., Peintinger M. F., Bredow T. (2019). BSSE-correction scheme
for consistent gaussian basis sets of double-and triple-zeta valence
with polarization quality for solid-state calculations. J. Comput. Chem..

[52] Botan-Neto B. D., Santamaria-Perez D., Wedek L., Bayarjargal L., Bera G., Botella P., Pellicer-Porres J., Otero-de-la-Roza A., Popescu C., Alabarse F. G. (2025). Compressibility
and Anisotropy of Trona: Unveiling the Structure of a Dense Na_3_H­(CO_3_)_2_·2H_2_O Polymorph. Inorg. Chem..

[53] Blatov V. A. (2006). Multipurpose
Crystallochemical Analysis with the Program Package TOPOS. Int. Union Crystallogr. Newsl..

[54] Blatov V. A., Shevchenko A. P., Proserpio D. M. (2014). Applied Topological Analysis of Crystal
Structures with the Program Package ToposPro. Cryst. Growth Des..

[55] Angel R. J. (2000). Equations
of state. Rev. Mineral. Geochem..

[56] Santamaría-Pérez D., Pavic L., Chulia-Jordan R., Ruiz-Fuertes J., Popescu C., Otero-de-la-Roza A. (2023). Phase stability of stress-sensitive
Ag_2_CO_3_ silver carbonate at high pressures and
temperatures. Solid State Sci..

[57] Gavryushkin P. N., Bekhtenova A., Lobanov S. S., Shatskiy A., Likhacheva A. Y., Sagatova D., Sagatov N., Rashchenko S. V., Litasov K. D., Sharygin I. S., Goncharov A. F., Prakapenka V. B., Higo Y. (2019). High-Pressure Phase Diagrams of Na_2_CO_3_ and K_2_CO_3_. Minerals.

[58] Holl C. M., Smyth J., Laustsen H., Jacobsen S., Downs R. T. (2000). Compression
of witherite to 8 GPa and the crystal structure of BaCO_3_-II. Phys. Chem. Miner..

[59] Vidal-Daza I., Sanchez-Navas A., Hernandez-Laguna A. (2023). Compressional behavior of the aragonite-structure
carbonates to 6 GPa. Phys. Chem. Miner..

[60] Chuliá-Jordán R., Santamaria-Perez D., Ruiz-Fuertes J., Otero-de-la-Roza A., Popescu C. (2021). Crystal Structure of
BaCa­(CO_3_)_2_ Alstonite Carbonate and Its Phase
Stability upon Compression. ACS Earth Space
Chem..

[61] Chuliá-Jordán R., Santamaría-Pérez D., González-Platas J., Otero-de-la-Roza A., Ruiz-Fuertes J., Popescu C. (2022). Phase stability and
dense polymorph of the BaCa­(CO_3_)_2_ barytocalcite
carbonate. Sci. Rep..

[62] Zhang J., Reeder R. J. (1999). Comparative compressibilities of
calcite-structure
carbonates: Deviations from empirical relations. Am. Mineral..

[63] Chuliá-Jordán R., Santamaria-Perez D., Otero-de-la-Roza A., Ruiz-Fuertes J., Marqueño T., Gomis O., MacLeod S., Popescu C. (2020). Phase stability
of natural Ni_0.75_Mg_0.22_Ca_0.03_CO_3_ gaspeite mineral at high pressure and temperature. J. Phys. Chem. C.

[64] Chuliá-Jordán R., Santamaria-Perez D., Ruiz-Fuertes J., Otero-de-la-Roza A., Popescu C. (2021). Compressibility and
phase stability of iron-rich ankerite. Minerals.

[65] Pereira A. L. J., Errandonea D., Beltrán A., Gracia L., Gomis O., Sans J. A., García-Domene B., Miquel-Veyrat A., Manjón F. J., Muñoz A., Popescu C. (2013). Structural study of
α-Bi_2_O_3_ under pressure. J. Phys.: Condens. Matter.

[66] Becke A. D., Edgecombe K. E. (1990). A simple
measure of electron localization in atomic
and molecular systems. J. Chem. Phys..

[67] Yu M., Trinkle D. R. (2011). Accurate and efficient
algorithm for Bader charge integration. J. Chem.
Phys..

[68] Otero-de-la-Roza A., Johnson E. R., Luaña V. (2014). Critic2: a program for real-space
analysis of quantum chemical interactions in solids. Comput. Phys. Commun..

[69] Bonales L. J., Muñoz-Iglesias V., Santamaria-Perez D., Caceres M., Fernandez-Remolar D., Prieto-Ballesteros O. (2013). Quantitative
Raman spectroscopy as a tool to study the kinetics and formation mechanism
of carbonates. Spectrochim. Acta.

[70] Santamaria-Perez D., Ruiz-Fuertes J., Peña-Alvarez M., Chulia-Jordan R., Marqueño T., Zimmer D., Gutierrez-Cano V., MacLeod S., Gregoryanz E., Popescu C., Rodrıguez-Hernandez P., Muñoz A. (2019). Post-tilleyite,
a dense calcium silicate-carbonate
phase. Sci. Rep..

[71] Taylor P., Sunder S., Lopata V. J. (1984). Structure, spectra, and stability
of solid bismuth carbonates. Can. J. Chem..

[72] Dong F., Lee S. C., Wu Z., Huang Y., Fu M., Ho W.-K., Zou S., Wang B. (2011). Rose-like monodisperse
bismuth subcarbonate hierarchical hollow microspheres: One-pot template-free
fabrication and excellent visible light photocatalytic activity and
photochemical stability for NO removal in indoor air. J. Hazard. Mater..

[73] Santamaría-Pérez D., Chulia-Jordan R., Botan-Neto B. D., Bera G., Pellicer-Porres J., Bayarjargal L., Otero-de-la-Roza A., Popescu C. (2025). Pressure-driven phase
transformations on Mg_3_Ca­(CO_3_)_4_ huntite
carbonate. Phys. Chem. Chem. Phys..

