# Supplementary material for: Structure and Compressional Behavior of Sillén-Type (BiO)2(CO3) Bismutite Carbonate
Source: Inorg Chem. 2026 Jul 24;65(31):17955–66. doi: 10.1021/acs.inorgchem.6c01891 (PMC13463688; doi:10.1021/acs.inorgchem.6c01891)
Supplement: Supplementary file 1 [file ic6c01891_si_001.pdf]

Supplementary Material

of

Structure and compressional behavior of Sillén-type $(BiO)_2(CO_3)$ bismutite carbonate

Raquel Chuliá-Jordán[1,*], David Santamaría-Pérez[2], Benedito Donizeti Botan-Neto[2], Ganesh Bera[2], Julio Pellicer-Porres, Alberto Otero-de-la-Roza[3], Lkhamsuren Bayarjargal[4], Julia Bungarten[4], Catalin Popescu[5], and Bihan Wang[6]

[1] *Departamento de Didáctica de las Ciencias Experimentales y Sociales, Universitat de Valencia, 46022, Valencia, Spain.* **email: Raquel.Chulia@uv.es.*

[2] *Departamento de Física Aplicada-ICMUV, Universitat de València, MALTA Consolider Team, 46100 Valencia, Spain.*

[3] *Departamento de Química Física y Analítica, Facultad de Química, Universidad de Oviedo, MALTA Consolider Team, 33006, Oviedo, Spain*

[4] *Institute of Geosciences, Goethe University Frankfurt, Frankfurt 60438, Germany*

[5] *CELLS-ALBA Synchrotron Light Facility, Cerdanyola del Vallés, 08290, Barcelona, Spain*

[6] *Deutsche Elektronen-Synchrotron DESY, Notkestraße 85, Hamburg 22607, Germany*

Table S1 *– Fractional atomic coordinates and equivalent isotropic displacement parameters for bismutite (U_{eq} is defined as one-third of the trace of the orthogonalized U_{ij} tensor).*

**Atom**	**Wyckoff Site**	**x**	**y**	**z**	**Occupancy**	**U_{eq} (Å^2)**
Bi1	8f	0.5000	0.34265(8)	0.50561(3)	1.0	0.0237(3)
Bi2	4c	0.5000	0.16744(9)	0.2500	1.0	0.0159(3)
Bi3	4c	0.0000	0.35151(9)	0.2500	1.0	0.0178(3)
C1	4c	0.5000	0.4930(30)	0.7500	1.0	0.051(17)
C2	4b	0.0000	0.5000	0.5000	1.0	0.007(2)
O1	16h	0.2562(10)	0.2595(8)	0.1273(7)	1.0	0.025(3)
O2	4c	0.5000	0.4012(18)	0.7500	1.0	0.022(2)
O3	16h	0.6350(20)	0.5446(19)	0.8235(12)	0.5	0.027(3)
O4	16h	-0.1550(60)	0.4490(30)	0.4380(30)	0.25	0.040
O5	16h	-0.0890(60)	0.5720(30)	0.4390(30)	0.25	0.040
O6	16h	0.1440(70)	0.4900(50)	0.5870(30)	0.25	0.040

Table S2 *- Anisotropic displacement parameters for bismutite (the anisotropic displacement factor exponent is expressed as:* $-2\pi^2[h^2a^{*2}U_{11} + 2hka^*b^*U_{12} + \ldots]$*).*

**Atom**	**U_{11} (Å²)**	**U_{22} (Å²)**	**U_{33} (Å²)**	**U_{23} (Å²)**	**U_{13} (Å²)**	**U_{12} (Å²)**
Bi1	0.0186(2)	0.0363(10)	0.01633(19)	-0.00130(17)	0.0000	0.0000
Bi2	0.0176(3)	0.0132(9)	0.0170(2)	0.0000	0.0000	0.0000
Bi3	0.0172(3)	0.0170(9)	0.0193(2)	0.0000	0.0000	0.0000
O1	0.022(2)	0.036(11)	0.018(2)	0.002(3)	0.0058(16)	0.000
C1	0.018(10)	0.070(60)	0.070(20)	0.0000	0.0000	0.0000

***Table S3** - Fractional atomic coordinates of the DFT-calculated monoclinic Pc $(BiO)_2CO_3$ phase at 0.23 GPa. Lattice parameters are a = 13.835 Å, b = 10.877 Å, c = 5.474 Å and β = 91.6º.*

Atom	Wyckoff Site	x	y	z	Occupancy
Bi1	2a	0.83258	0.24643	0.53097	1.0
Bi2	2a	0.33492	0.24367	0.97836	1.0
Bi3	2a	0.16813	0.74542	0.46866	1.0
Bi4	2a	0.66450	0.74313	0.02503	1.0
Bi5	2a	0.64870	0.25651	0.00822	1.0
Bi6	2a	0.15081	0.25733	0.45247	1.0
Bi7	2a	0.35174	0.75703	0.98260	1.0
Bi8	2a	0.84834	0.75775	0.54757	1.0
O1	2a	0.59648	0.74966	0.53297	1.0
O2	2a	0.09065	0.76507	0.03162	1.0
O3	2a	0.40308	0.24418	0.51000	1.0
O4	2a	0.91030	0.26976	0.96635	1.0
O5	2a	0.73516	0.13081	0.75217	1.0
O6	2a	0.23863	0.12644	0.21651	1.0
O7	2a	0.26459	0.62367	0.23391	1.0
O8	2a	0.76057	0.62851	0.79046	1.0
O9	2a	0.74490	0.37125	0.27993	1.0
O10	2a	0.25069	0.37602	0.74079	1.0
O11	2a	0.25179	0.87556	0.73144	1.0
O12	2a	0.75279	0.87137	0.26849	1.0
C1	2a	0.50300	0.74753	0.49999	1.0
C2	2a	0.99854	0.75229	0.06692	1.0
C3	2a	0.49665	0.24969	0.49494	1.0
C4	2a	0.00213	0.25018	0.93772	1.0
O13	2a	0.93694	0.82036	0.94950	1.0
O14	2a	0.54386	0.32983	0.62337	1.0
O15	2a	0.06582	0.31685	0.05179	1.0
O16	2a	0.97115	0.67377	0.22674	1.0
O17	2a	0.53922	0.17545	0.34917	1.0
O18	2a	0.02724	0.16644	0.78675	1.0
O19	2a	0.46545	0.66907	0.34937	1.0
O20	2a	0.45062	0.82520	0.61529	1.0

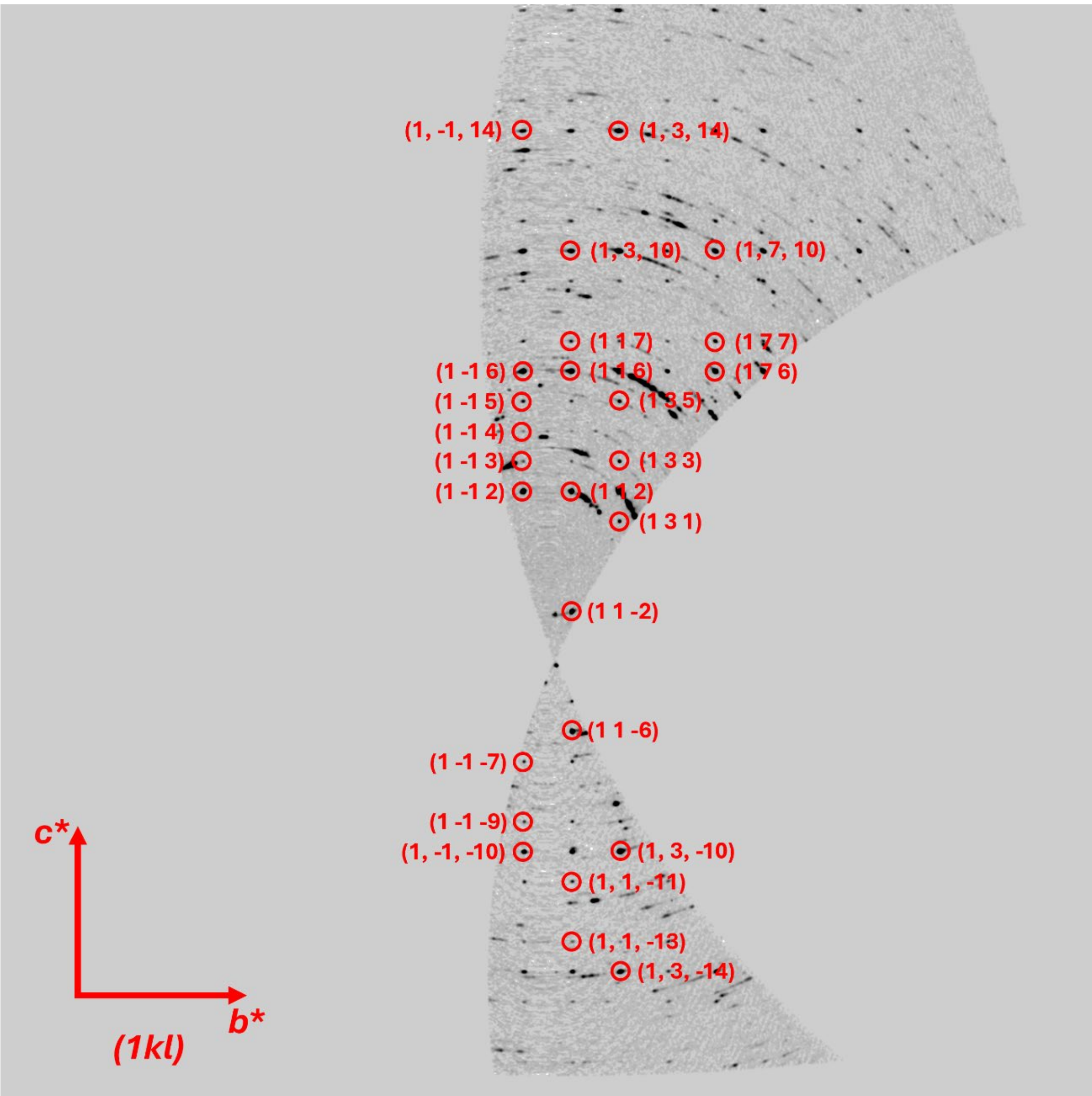

Figure S1.- Reconstructed reciprocal-space precession image of the (1kl) plane for *Cmcm* bismutite at ambient conditions. Selected reflections are highlighted with red circles and labeled with their corresponding Miller indices.

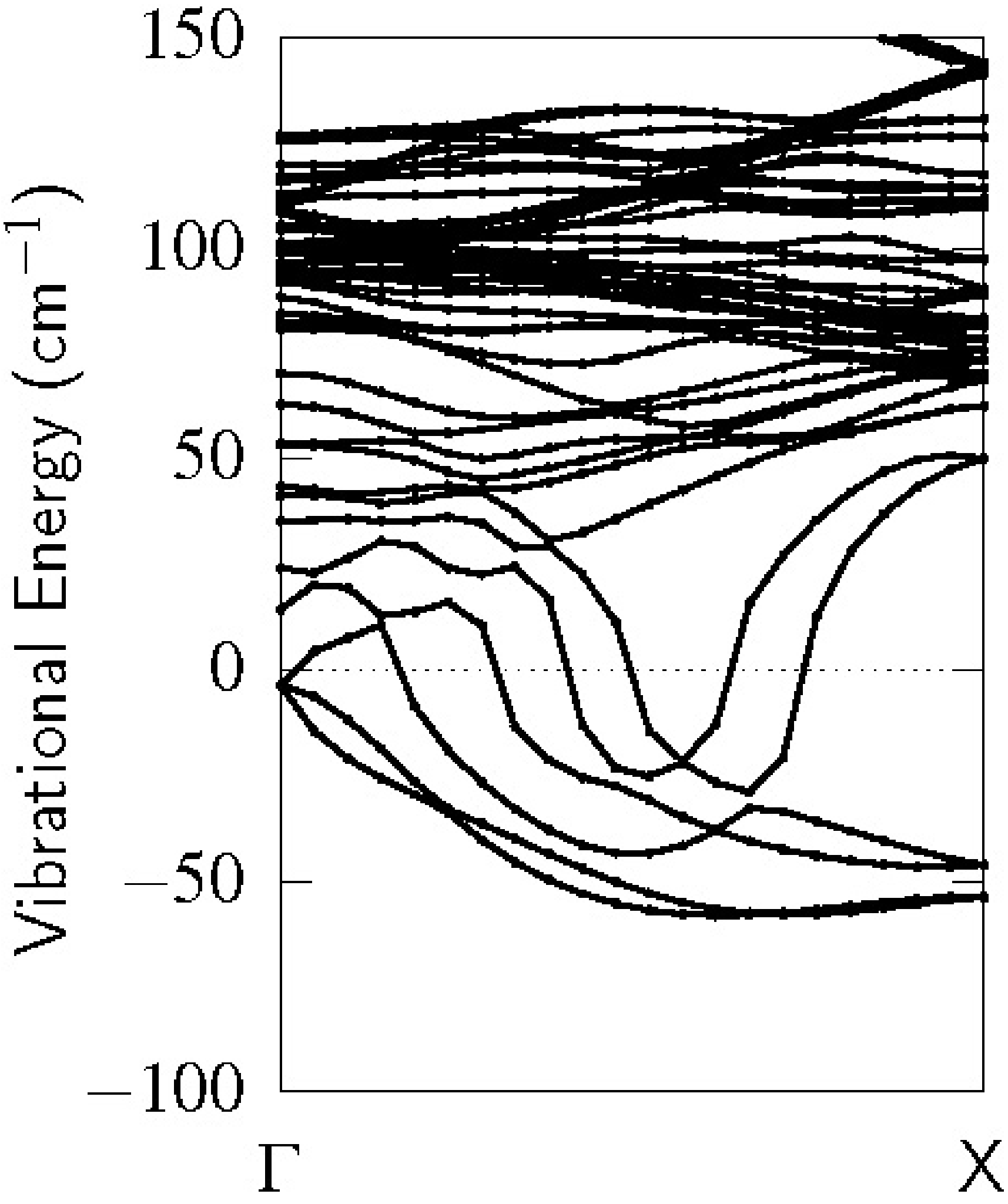

Figure S2.- Vibrational band structure plot of the *Pna*2_1 structure at 20.9 GPa in the Γ to X direction.

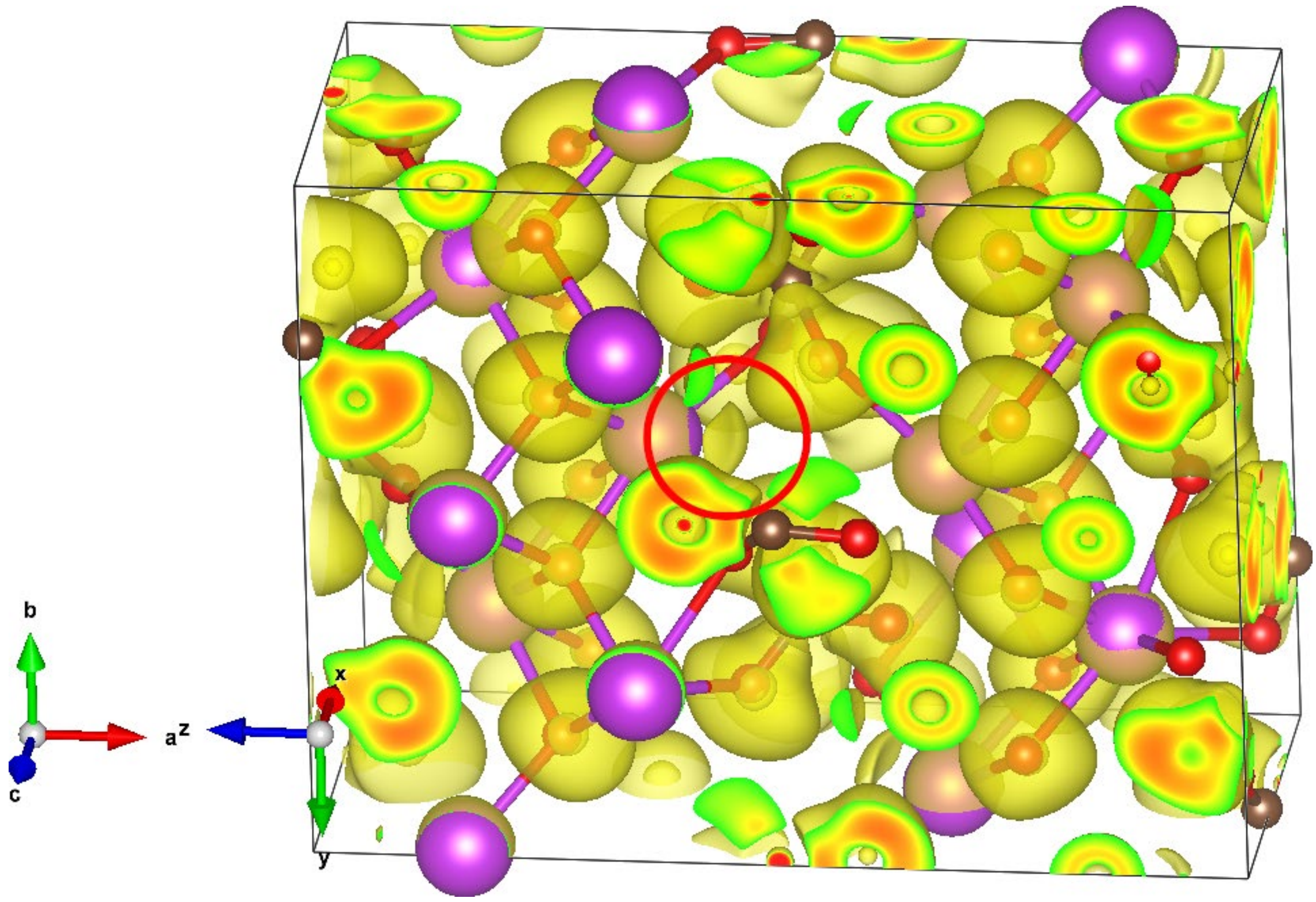

Figure S3.- Representation of the isosurfaces of the electron localization function (ELF) in bismutite, where a position of the ELF maximum corresponding to the Bi lone pair has been encircled in red.

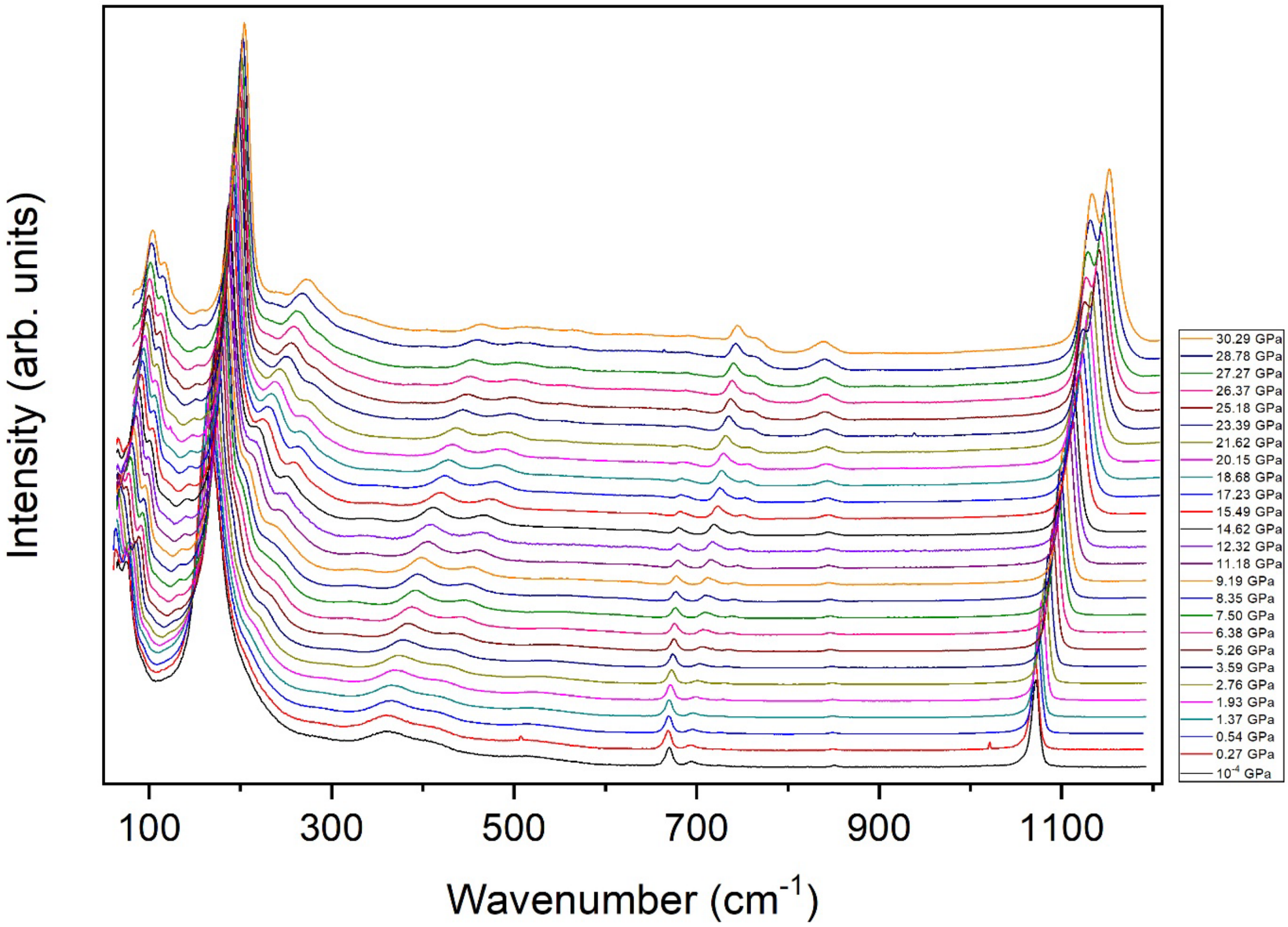

Figure S4.- Room-temperature Raman spectra of $(BiO)_2CO_3$ bismutite on pressure upstroke in the frequency region 60–1200 cm^{-1}.

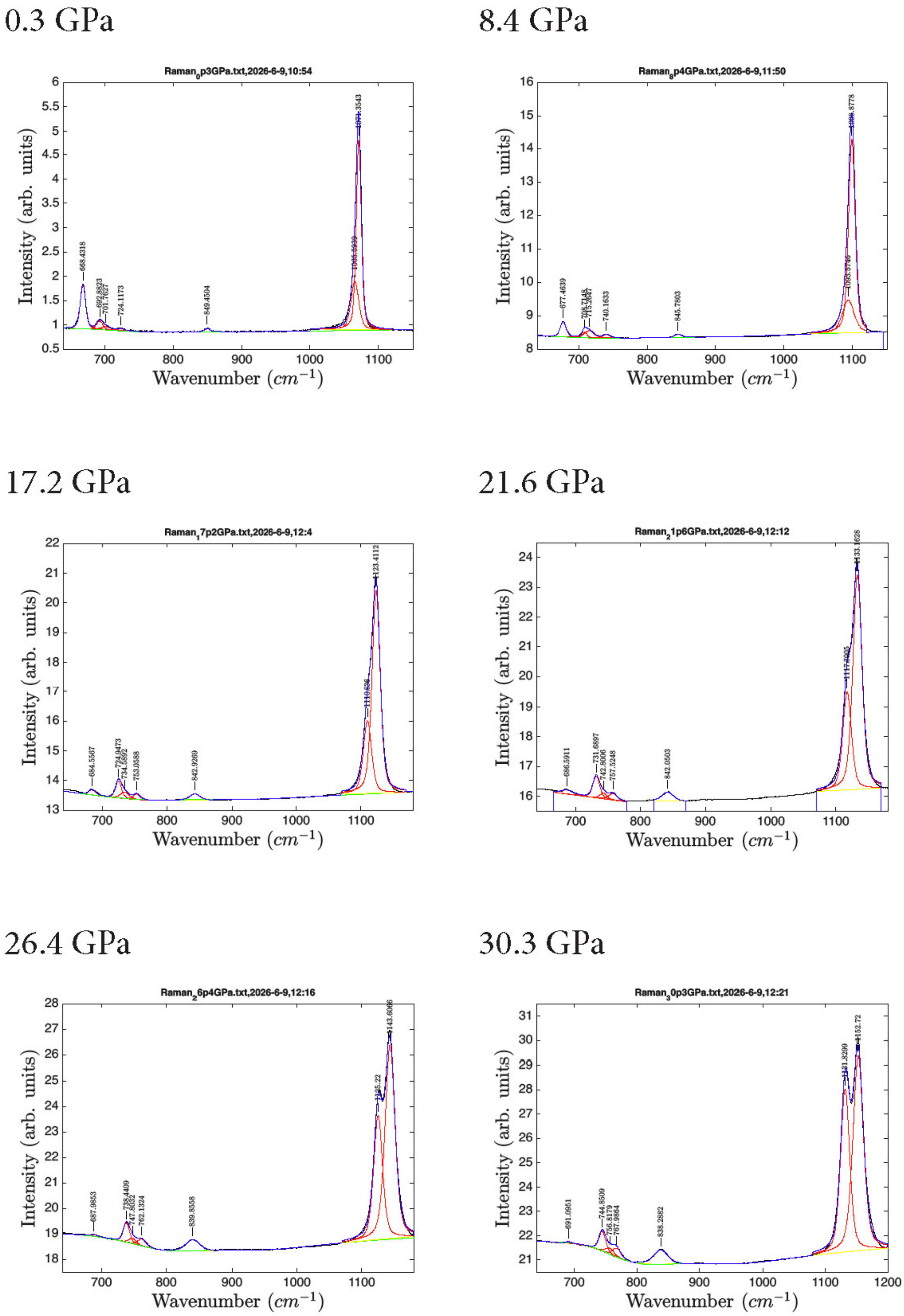

Figure S5.- Raman peak-fitting analyses at selected pressures.
